# Integrating zinc homeostasis network and immune landscape: a five-gene prognostic framework for precision oncology in lung adenocarcinoma

**DOI:** 10.3389/fimmu.2025.1691179

**Published:** 2026-01-08

**Authors:** Feng Cheng, Jinhe Xu, Wenting Zhang, Xinyu Zhang, Ying Chen, Nong Zhou, Chenxin Li, Zongyang Yu

**Affiliations:** 1 Fuzong Clinical Medical College of Fujian Medical University, Fuzhou, Fujian, China; 2 Fuzong Teaching Hospital of Fujian University of Traditional Chinese Medicine (900th Hospital), Fuzhou, China; 3 Department of Pulmonary and Critical Care Medicine, Fuzong Clinical Medical College of Fujian Medical University and 900th Hospital of PLA Joint Logistic Support Force, Fuzhou, Fujian, China

**Keywords:** immunotherapy, LUAD, prognostic model, tumor microenvironment, zinc homeostasis network

## Abstract

**Background:**

Lung adenocarcinoma (LUAD) exhibits marked heterogeneity in clinical outcomes and therapeutic responses, underscoring the imperative for reliable prognostic biomarkers. Dysregulation of zinc homeostasis is an emerging hallmark of cancer, contributing to tumor progression through multifaceted mechanisms including exacerbated oxidative stress and sustained oncogenic signaling. This study aimed to develop and validate a novel prognostic signature based on zinc homeostasis network-related genes for stratifying LUAD patients into distinct risk groups to predict clinical outcomes and inform therapeutic strategies.

**Methods:**

Transcriptomic and clinical profiles of LUAD cases from the TCGA database were integrated to screen for differentially expressed genes (DEGs) involved in zinc homeostasis network. A prognostic risk model was constructed via univariate, multivariate, and LASSO regression analyses, and externally validated using GEO datasets. Model performance was evaluated using a nomogram, time-dependent ROC curves, and decision curve analysis. To characterize immune microenvironment heterogeneity across risk subgroups, we applied seven deconvolution algorithms, ssGSEA and single-cell profiling. Spearman correlation analysis and Wilcoxon rank-sum tests were used for investigating the associations between risk stratification and immunomodulatory markers, tumor mutational burden (TMB), as well as the predicted responsiveness to conventional chemotherapeutic and targeted therapies. *In vitro* experiments validated the expression levels of key candidate genes and confirmed their biological functions.

**Results:**

Differential expression analysis identified 124 zinc homeostasis network-related DEGs, of which 16 showed significant prognostic relevance. A five-gene risk model stratified patients into distinct prognostic groups, with high-risk cases showing markedly reduced overall survival. Risk score correlated positively with advanced clinical stages. Multivariate Cox regression confirmed the model as independently predictive of LUAD prognosis. A nomogram integrating risk score and clinical features was constructed for predicting 1-, 3-, and 5-year survival. Immune profiling showed that low-risk cases had hot tumor phenotypes, elevated immune scores and infiltration by immune cells, while those at high risk showed raised levels of immunotherapy resistance markers and increased TMB. Drug sensitivity analysis indicated differential responses to chemotherapeutic and targeted agents across risk groups. Independent knockdown of SLC16A3 or overexpression of EGR2 significantly suppressed malignant behaviors in LUAD cells. Additionally, SLC16A3 downregulation reduced cisplatin sensitivity in LUAD cells.

**Conclusion:**

Our study highlights the clinical translational potential of a five-gene zinc homeostasis network signature in predicting prognosis and guiding personalized therapeutic strategies for LUAD.

## 1 Introduction

Among all types of lung cancer, lung adenocarcinoma (LUAD) is a prevalent and aggressive non-small cell lung cancer subtype, accounting for nearly 40% of cases and exhibiting marked intertumoral heterogeneity (1). Despite advancements in targeted therapies and immune checkpoint inhibitors, over 60% of patients develop therapeutic resistance within 12 months (2–4). This high rate of treatment failure underscores the critical unmet need for novel prognostic frameworks to both predict clinical outcomes and guide personalized therapeutic decisions.

Zinc ions function as essential intracellular second messengers (5), with tightly regulated homeostasis required for key physiological processes, including cellular growth, development, and immune regulation (6–8). Increasing evidence indicates that zinc homeostasis dysregulation is a hallmark of tumor progression (9, 10), wherein aberrant zinc flux drives tumor progression by activating oncogenic pathways such as MAPK and Akt to promote oncogenesis (11–13), while simultaneously inducing epithelial-mesenchymal transition (EMT) and enhancing matrix metalloproteinase-driven extracellular matrix degradation to facilitate invasiveness and metastasis (9, 14). Proteins involved in zinc transport serve as critical regulators of intracellular zinc homeostasis, and dysregulation of their expression has been linked to multiple malignant phenotypes. For instance, Yang et al. demonstrated that ZIP4 overexpression accelerates pancreatic tumor growth and cachexia in mice by stimulating RAB27B-mediated extracellular vesicle production (15). Similarly, Franklin et al. reported that reduced expression of hZIP1 contributes to early prostate cancer progression (16). Although zinc homeostasis network dysregulation promotes malignant progression in various cancers and specific transporters like SLC39A4 are implicated in lung cancer pathogenesis (17, 18), their collective impact on LUAD immune microenvironment remodeling remains uncharacterized. The potential of multi-gene signatures for constructing prognostic models and guiding clinical therapeutic regimens remains unexplored. To address these gaps, we propose to develop an integrated prognostic model based on zinc homeostasis network to provide insights into novel therapeutic approaches for LUAD.

In this study, zinc homeostasis network-associated hub genes were identified and analyzed in LUAD, and their potential roles in tumor progression were elucidated through functional enrichment analysis. A model incorporating five signature genes was constructed and validated for predicting prognosis employing multivariate Cox regression and machine learning algorithms. We further assessed the model’s association with the tumor immune landscape and its potential in predicting responses to chemotherapy and targeted agents. Taken together, these findings offer a novel framework for personalized therapeutic decision-making and prognostic marker selection in LUAD, shedding new light on the role of zinc homeostasis network in disease progression.

## 2 Materials and methods

### 2.1 Data acquisition and analysis of zinc homeostasis network-related differentially expressed genes

RNA-seq datasets in TPM format, along with matched clinical data for 541 LUAD tumors and 59 adjacent normal tissues were retrieved from TCGA. Wilcoxon rank-sum tests were utilized to identify differentially expressed genes (DEGs), with thresholds set at false discovery rate (FDR) < 0.05 and |log_2_ fold change| > 1.0, using a volcano plot for visualization. Zinc homeostasis network-related gene sets were sourced from GeneCards (https://www.genecards.org/) using the search term “zinc transport”, as these transporters represent the primary regulators of intracellular zinc homeostasis, and 124 zinc homeostasis network-related DEGs were determined via Venn diagram intersection. Protein-protein interaction (PPI) networks were constructed using STRING (http://string-db.org) and visualized with Cytoscape. To define a highly interconnected gene cluster suitable for functional enrichment analysis, the top 60 hub genes were selected using the Maximal Clique Centrality (MCC) algorithm within the CytoHubba plugin for Cytoscape.

### 2.2 Prognostic risk model construction and validation in LUAD

LUAD cases with overall survival <30 days were excluded to minimize immortal time bias. Sixteen genes linked to prognosis were identified in the univariate analysis (p < 0.05). Subsequent integration of LASSO regression and multivariate Cox analysis refined the selection to five genes demonstrating significant prognostic value. These genes were used to establish a zinc homeostasis network-related gene signature for risk stratification. The risk score was calculated as follows:


Risk Score=Σ(expression level of each gene×corresponding regression coefficient).

Cases were assigned to high-risk (HR) and low-risk (LR) groups based on the median risk score. Kaplan-Meier (K-M) survival curve and time-dependent Receiver Operating Characteristic (ROC) curve analyses were conducted using the “survival” and “timeROC” R packages. Dimensionality reduction and visualization were performed using Principal Component Analysis (PCA) and t-distributed Stochastic Neighbor Embedding (t-SNE) with the “limma,” “Rtsne,” and “ggplot2” R packages. Model performance was further validated in three independent GEO cohorts: GSE26939, GSE72094, and GSE13213, along with corresponding clinicopathological features and survival outcomes derived from the GEO database.

### 2.3 Assessment of immune microenvironment

Lymphocyte infiltration in HR and LR groups was profiled using the “IOBR” R package (19), with group differences visualized via heatmap. Seven established deconvolution algorithms-including XCELL, TIMER, QUANTISEQ, MCP-COUNTER, EPIC, CIBERSORT-ABS, and CIBERSORT-were employed to comprehensively evaluate correlations of risk score with immune cell subpopulations in the tumor microenvironment (TME). Immune function across groups was systematically analyzed using the “ssGSEA” algorithm. TME characteristic scores, including immune score, stroma score, and ESTIMATE score, were determined with the “estimate,” “reshape2,” and “ggpubr” R packages. For immunotherapy response prediction, intergroup comparisons of TIDE Score, CAF Score, MDSC Score, and Exclusion Score were facilitated by the R packages “ggplot2,” “ggpubr,” and “tidyverse”.

### 2.4 Human tissues and cells

The LUAD tissue and adjacent lung tissue samples used in this study were all obtained from five primary patients who had signed informed consent forms, with samples collected from the 900th Hospital of the Joint Logistic Support Force of China. According to the Declaration of Helsinki, this study has been approved by the Ethics Committee of the 900th Hospital of the Joint Logistic Support Force of China (Approval No.: 2025-047). Human LUAD cell lines A549 and H1975 were purchased from the Cell Bank of the Shanghai Institute of Biological Sciences (Shanghai, China). All cells were cultured in a humidified incubator at 37°C with 5% CO_2_. A549 cells were cultured in DMEM/F-12 medium containing 10% fetal bovine serum (FBS), and H1975 cells were cultured in RPMI 1640 medium containing 10% FBS.

### 2.5 Cell transfection

Cell transfection experiments were performed using lipid nanoparticle-based reagents (GA-RNA Transfection Kit and GA-DNA Transfection Kit, GeneAdv Co. Ltd, Suzhou, China). SLC16A3 expression was knocked down using two different types of siRNA, with the following sequences:

si-SLC16A3#1: GGAGCAUCAUCCAGGUCUATT,
si-SLC16A3#2: CCGUCAGUGUCUUCUUCAATT.

Cells transfected with nonspecific siRNA (si-con) served as the negative control. For EGR2 overexpression, the pCMV-C-3xFLAG-h-EGR2 plasmid and its corresponding empty vector control were used. Transfection efficiency was assessed by Western blot 48 hours after transfection.

### 2.6 Cellular function assays

For CCK-8 viability assays, cells were seeded in 96-well plates at 1,000 cells/well. After 72 hours incubation, cell viability was assessed by measuring the optical density at 450 nm using a microplate reader following incubation with CCK-8 reagent (Meilunbio, Dalian, China) for 1.5 hours at 37°C. For EdU proliferation assays, cells were plated in 24-well plates at 2×10^4^ cells/well and cultured for 72 hours. Cells were fixed with 4% paraformaldehyde for 30 min and permeabilized with Triton X-100 for 15 min before staining according to the EdU kit protocol (Beyotime, Shanghai, China), and the percentage of EdU positive nuclei was determined from three randomly selected fields under a fluorescence microscope. Cell migration was evaluated using “Transwell” chambers with 8-μm pores. Briefly, cells resuspended in serum-free medium were seeded into the upper chamber at 3.5×10^4^ cells/chamber, while the lower chamber contained 0.6 mL of 20% serum-supplemented medium. After 24 hours incubation, migrated cells on the lower membrane surface were fixed with 4% paraformaldehyde for 30 min, stained with 2.5% crystal violet for 20 min, and counted in three random fields per chamber under an inverted microscope. These functional assay protocols have been validated in previous studies (20, 21).

### 2.7Cisplatin sensitivity and apoptosis assays

The GSE213102, GSE222187, and GSE108214 datasets were downloaded from the GEO database, and *SLC16A3* expression levels were extracted and compared between cisplatin-sensitive and cisplatin-resistant LUAD cell lines. For the *in vitro* drug sensitivity assay, A549 cells were transfected with si-con or si-SLC16A3#1. At 48 hours post-transfection, cells were seeded into 96-well plates at equal densities, and then treated with increasing concentrations of cisplatin (Meilunbio, Dalian) for an additional 24 hours. Cell viability was measured using the CCK-8 assay, and the IC50 value for cisplatin was calculated from the dose-response curve. To evaluate cisplatin-induced apoptosis, A549 and H1975 cells were transfected with si-con or SLC16A3 siRNA. For the TUNEL assay, cells were treated with 10 μM cisplatin for 24 hours at 48 hours after transfection, followed by staining using the TUNEL Apoptosis Detection Kit (Beyotime, Shanghai). Images were captured under a fluorescence microscope, and the percentage of TUNEL-positive cells was quantified. For Annexin V/PI flow cytometry, cells were similarly treated with 10 μM cisplatin for 24 hours at 48 hours post-transfection. Cells were collected, washed with PBS, and stained using the Annexin V-FITC/PI Apoptosis Detection Kit (Meilunbio, Dalian). After incubation at room temperature in the dark for 10–15 minutes, samples were analyzed by flow cytometry, and the proportions of apoptotic cells were determined using FlowJo software.

### 2.8 Statistical analysis

All bioinformatics analyses were conducted using the R (version 4.3.3). Functional assays included three independent biological replicates, with data expressed as mean ± SD. Statistical analysis used GraphPad Prism 7.0, with group differences assessed by one-way ANOVA or Student’s t-test. Statistical significance was defined as p < 0.05.

## 3 Results

### 3.1 Identification of zinc homeostasis network-related DEGs between normal and LUAD tissues

**Figure 1:**
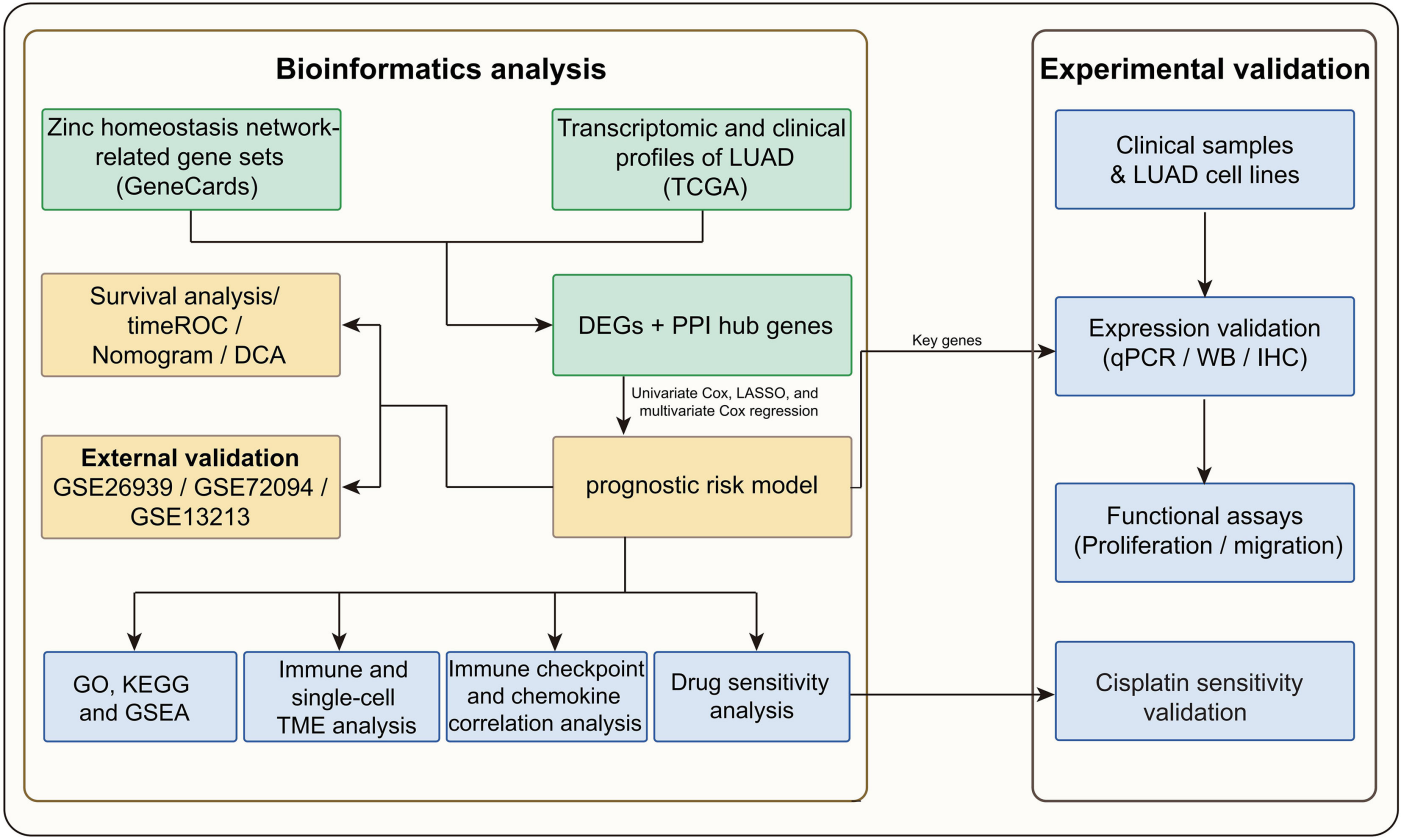
Flowchart of the study.

**Figure 2:**
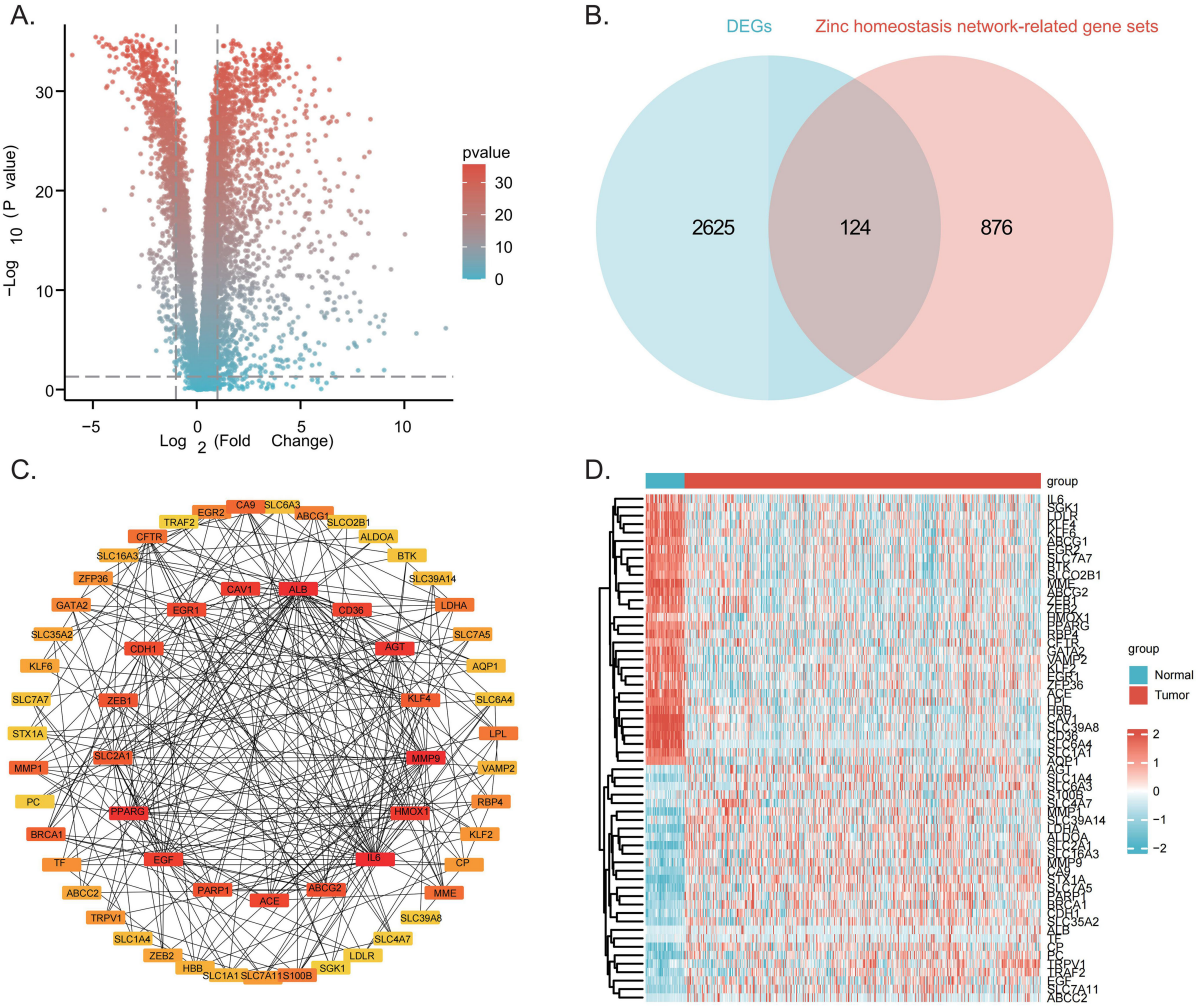
Identification of zinc homeostasis network-related DEGs between normal and LUAD tissues. **(A)** Volcano plot showing the distribution patterns of DEGs between LUAD and normal tissues. **(B)** Venn analysis illustrating the intersection of DEGs with zinc homeostasis network-related gene sets. **(C)** PPI network of 60 hub genes. **(D)** Heatmap depicting the differential expression profiles of 60 hub genes between tumor and adjacent normal tissues.

The overall workflow of this study is illustrated in **Figure 1**. Comparison of the transcriptomic data of LUAD and normal tissue pairs identified 2,749 DEGs, of which 1,868 were upregulated and 881 were downregulated. These are illustrated in the volcano plot (**Figure 2A**). Intersection analysis with zinc homeostasis network-related gene sets indicated 124 zinc homeostasis network-associated DEGs (**Figure 2B**). To pinpoint core regulators, a PPI network was generated, from which 60 hub genes were identified using topological analysis (**Figure 2C**). These hub genes included transcriptional regulators (e.g., *PPARG*, *EGR2*), metabolism-related genes (e.g., *SLC16A3*, *LDHA*), and transmembrane signaling molecules (e.g., *CFTR*, *CAV1*). The expression levels of hub genes in tumor versus normal tissues were visualized through heatmap analysis (**Figure 2D**).

### 3.2 GO and KEGG

To evaluate functional roles and signaling pathways associated with zinc homeostasis network-related DEGs, GO and KEGG enrichment analyses were performed on the 60 hub genes, incorporating both standard and log_2_FC-weighted methods. GO enrichment covered BP, CC, and MF, revealing significant associations with key physiological functions such as hypoxia response, glucose homeostasis, lipid localization, among others (**Supplementary Figures S1A, B**). KEGG pathway analysis identified significant enrichment in oncogenic pathways, especially the PPAR and HIF-1 signaling pathways (**Supplementary Figure S1C**), suggesting potential molecular mechanisms by which zinc homeostasis network**-**related genes contribute to LUAD initiation and progression. These results were further supported by log_2_FC-weighting (**Supplementary Figure S1D**).

### 3.3 Construction of zinc homeostasis network-related prognostic risk model

**Figure 3:**
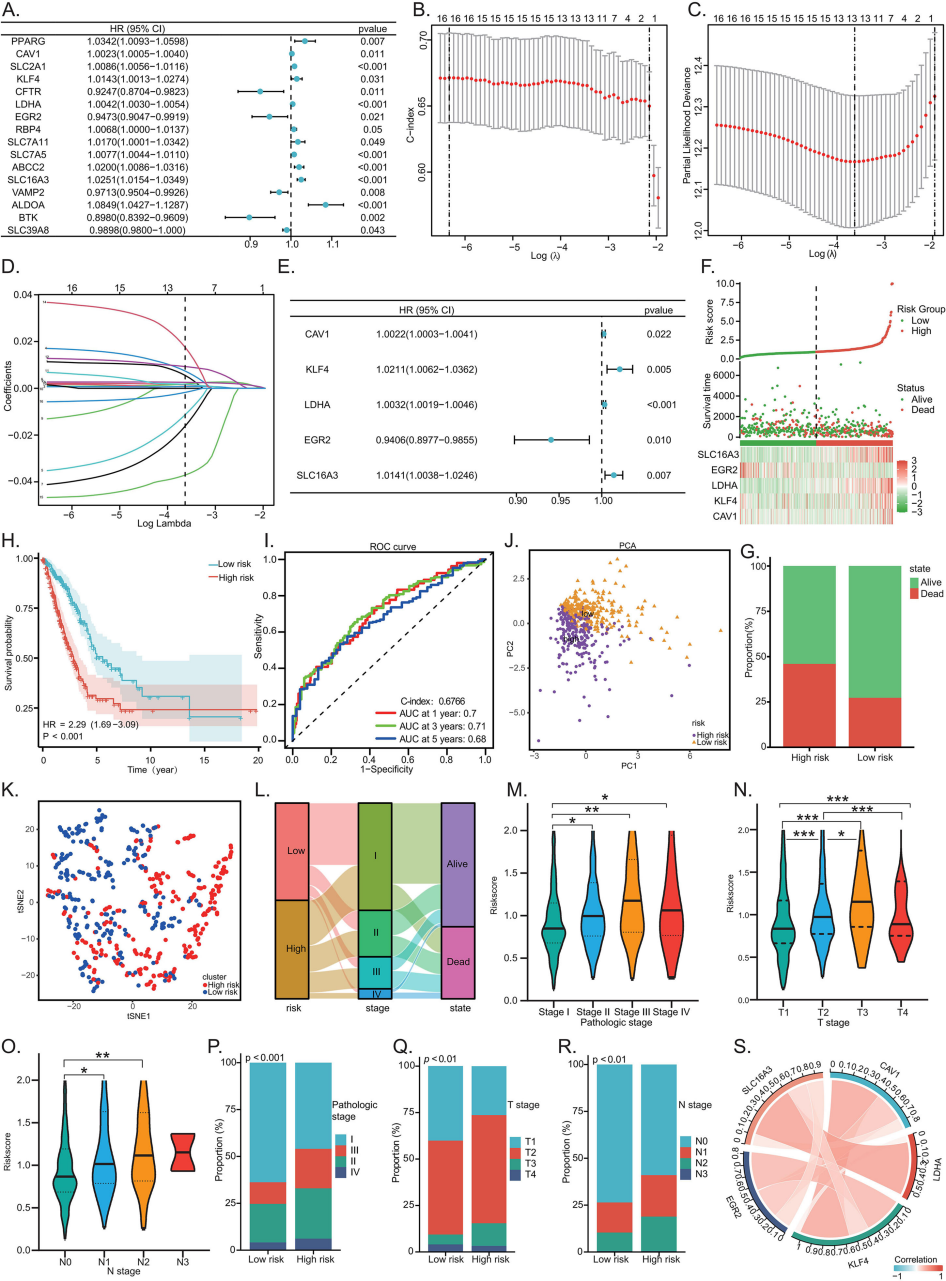
Construction of zinc homeostasis network-related prognostic risk model. **(A)** Univariate Cox regression analysis to identify zinc homeostasis network-related prognostic gene. LASSO regression analysis **(B-D)** and multivariate Cox regression analysis **(E)**. **(F)** Risk score distribution plot based on the median risk score. **(G)** Bar plot comparing mortality rates between HR and LR groups. **(H)** Kaplan-Meier survival curves (HR = 2.29, 95% CI: 1.69-3.09, log-rank p < 0.001). **(I)** ROC curves to evaluate predictive efficacy for 1-, 3-, and 5-year survival. PCA analysis **(J)** and t-SNE analysis **(K)**. **(L)** Sankey diagram illustrating the relationship among risk groups, pathological stages, and survival status. Differential analysis of risk score across clinicopathological subgroups: Pathological stage **(M)**, T stage **(N)**, N stage **(O)**. Composition of clinicopathologic subgroups in high- and LR groups: Pathological stage **(P)**, T stage **(Q)**, N stage **(R)**. **(S)** Interaction network of risk genes in LUAD. (*p < 0.05, **p < 0.01, ***p < 0.001).

Univariate Cox analysis of 60 hub genes identified 16 genes significantly associated with LUAD prognosis (**Figure 3A**), including key regulatory genes such as *PPARG*, *CAV1*, and *LDHA*. LASSO regression (**Figures 3B–D**) followed by multivariate Cox analysis (**Figure 3E**) yielded a final prognostic model consisting of five risk genes: *CAV1*, *KLF4*, *LDHA*, *EGR2*, and *SLC16A3*. Patients were stratified into HR and LR groups based on the median risk score. The risk factor analysis demonstrated a significant inverse correlation between risk score and clinical outcomes in the TCGA-LUAD cohort (**Figures 3F, G**). K-M survival analysis revealed significantly elevated mortality in the high-risk group (**Figure 3H**), with HR = 2.29 (95% CI: 1.69-3.09). The predictive ability of the model at 1-, 3-, and 5-year intervals was supported by time-dependent ROC curves, and the C-index of the five-gene signature was 0.6766 in the TCGA cohort (**Figure 3I**). Moreover, the AUC values of our integrated model surpassed those of any individual risk gene (**Supplementary Figures S2A–E**). PCA and t-SNE analyses confirmed separation of the groups (**Figures 3J, K**). A sankey diagram of clinicopathological correlations indicated a prevalence of early-stage (I-II) tumors in LR cases (**Figure 3L**). Subgroup analysis showed marked positive relationships between risk score and higher pathological, T, and N stages (**Figures 3M–O**). Furthermore, HR cases had markedly higher proportions of advanced-stage tumors compared to LR patients (**Figures 3P–R**). A chord diagram illustrates a functional cooperative network among the five risk genes during LUAD progression (**Figure 3S**), suggesting their biological interactions may contribute to disease advancement.

### 3.4 Construction of a prognostic nomogram model

**Figure 4:**
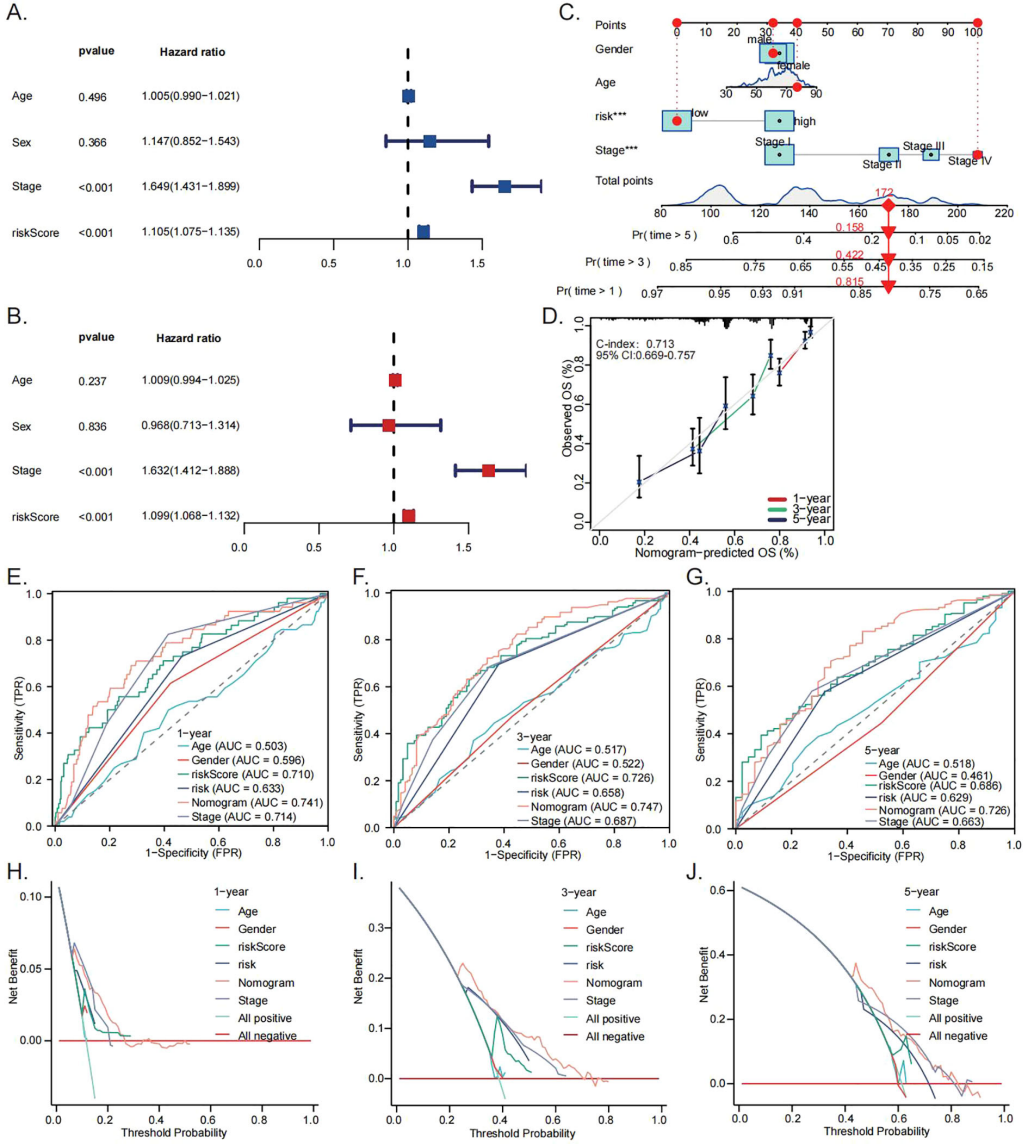
Construction of prognostic nomogram model. Univariate **(A)** and multivariate **(B)** Cox analyses assessing the prognostic significance of the risk score. Nomogram-based survival prediction for LUAD patients using the risk score **(C)**. Calibration curve associated with the nomogram **(D)**. ROC curves **(E–G)** and DCA **(H–J)** comparing the clinical benefit of the nomograms, risk score, and established clinical parameters at 1-, 3-, and 5-year follow-up intervals. (***p < 0.001).

According to Univariate Cox analysis, a significant positive correlation was observed between the risk score and unfavorable prognosis (**Figure 4A**). This association remained statistically significant after adjusting for potential confounders in multivariate analysis, supporting the role of the risk score as an independent prognostic indicator (**Figure 4B**). A nomogram integrating the risk score with clinicopathological features showed improved prognostic performance, with a C-index of 0.713 (**Figure 4C**). Calibration curves indicated excellent agreement between predicted and observed survival rates at 1, 3, and 5 years (**Figure 4D**). Time-dependent ROC analyses (**Figures 4E–G**) and decision curve analyses (**Figures 4H–J**) demonstrated that the nomogram outperformed individual clinical parameters in clinical net benefit across 1- to 5-year prediction periods, underscoring its potential utility for prognostic evaluation.

### 3.5 External validation of the prognostic risk model

**Figure 5:**
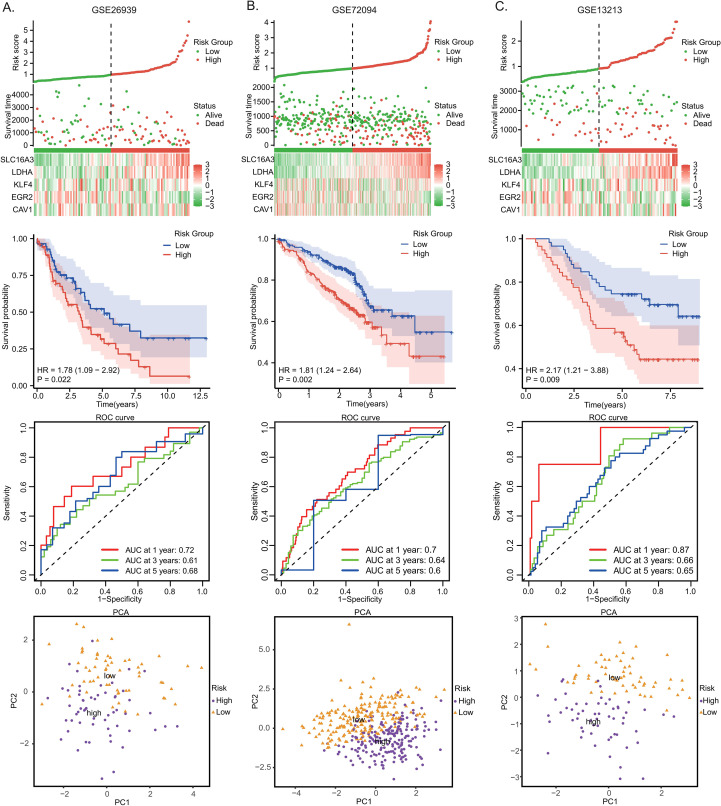
External validation of the prognostic risk model. External validation of the prognostic model: **(A)** GSE26939, **(B)** GSE72094, and **(C)** GSE13213.

In the GSE26939 (**Figure 5A**), GSE72094 (**Figure 5B**), and GSE13213 (**Figure 5C**) datasets, the risk factor distribution, K-M survival curves, and ROC analyses consistently aligned with findings from the TCGA-LUAD cohort, demonstrating that patients in the HR group displayed significantly poorer prognoses. The PCA further supported the reliability of the risk stratification, confirming clear separation between high- and LR groups. In addition, the C-index values of the five-gene signature in the GSE26939, GSE72094, and GSE13213 cohorts were 0.6379, 0.6513, and 0.6441, respectively, indicating a relatively stable discriminative ability across independent validation sets.

### 3.6 The potential molecular mechanism of the prognostic risk model

This study further delineated the biological functions and signaling pathways distinguishing high- and LR groups. GO analysis revealed that DEGs were primarily enriched in BP related to mitotic regulation and cell adhesion modulation. At the CC level, DEGs were concentrated in cell-substrate junction and focal adhesion. MF analysis further highlighted growth factor binding and MHC class II protein complex interactions as key features (**Supplementary Figure S3A**). KEGG pathway heatmap based on GSVA revealed preferential activation of pathways involving ATPase inhibitor, steroid hydroxylase, and related pathways in the LR group, whereas the HR group displayed enrichment in glucose catabolic and glycolytic process, as well as cadherin binding pathways (**Supplementary Figure S3B**). GSEA identified activation of the glycolysis and gluconeogenesis in the HR group, indicative of metabolic reprogramming (**Supplementary Figure S3C**), while the LR group showed selective activation of Th1/Th2 cell differentiation pathways (**Supplementary Figure S3D**). These integrative analyses provide mechanistic insights into the biological divergence between risk subtypes and offer a conceptual basis for identifying actionable therapeutic targets in LUAD.

### 3.7 Assessment of TME and immunotherapy

**Figure 6:**
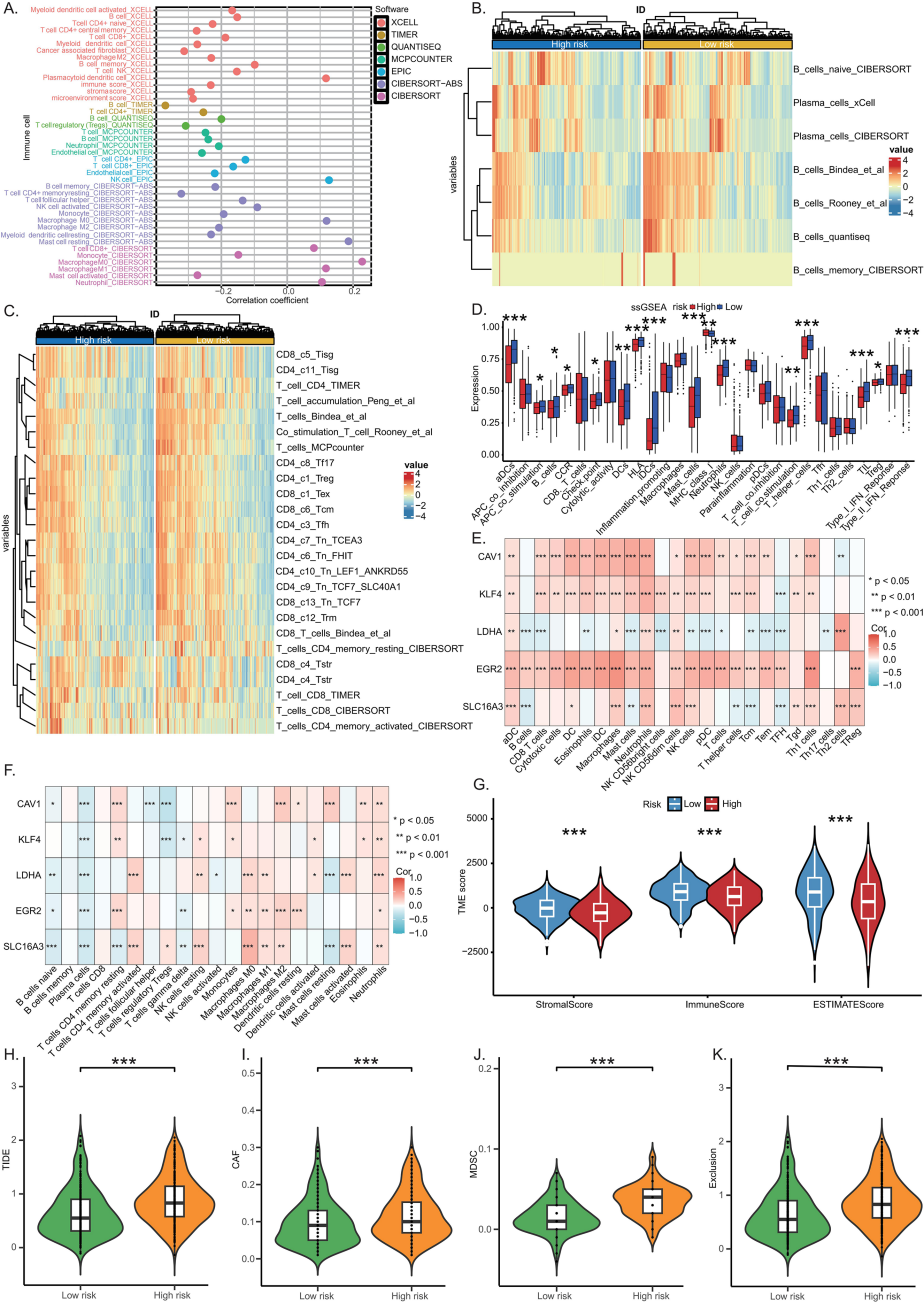
Assessment of TME and immunotherapy. **(A)** Overview of immune cell infiltration analysis across various algorithms. **(B, C)** Heatmap depicting differential infiltration levels of B lymphocytes and T lymphocytes between HR and LR groups. **(D)** Differential activation of immune functions between the groups. **(E, F)** Correlation analysis between independent risk genes and immune cell infiltration. **(G)** TME scores: stromal score, immune score, and estimate score. **(H)** TIDE score. **(I)** CAF score. **(J)** MDSC score. **(K)** Exclusion score. (*p < 0.05, **p < 0.01, ***p < 0.001).

Heatmap visualization integrated with immune cell infiltration data derived from multiple algorithms revealed a higher abundance of effector immune cells, including B lymphocytes and various T cell subsets, within the TME of LR LUAD patients compared to their HR counterparts (**Figures 6A-C**). Immune function analysis using ssGSEA further indicated increased activity of pathways associated with the immune systems in the LR group, particularly in antigen-presenting dendritic cells (aDCs), Type II interferon response, T helper cells, neutrophils, and immature dendritic cells (iDCs) (**Figure 6D**). Furthermore, both ssGSEA and CIBERSORT analyses revealed strong associations between the identified risk genes’ expression levels and distinct immune signatures (**Figures 6E, F**). The LR group also showed significantly elevated stromal score, immune score, and estimate score (**Figure 6G**), consistent with a “hot tumor” phenotype and elevated immunotherapy responsiveness. The HR group showed increased expression of biomarkers associated with immune evasion and therapy resistance, including elevated TIDE, CAF, MDSC, and Exclusion score (**Figures 6H-K**), suggestive of inherent resistance to immune checkpoint blockade. These findings support the utility of the risk model in delineating immune landscape heterogeneity and predicting immunotherapeutic response in LUAD.

### 3.8 Single-cell dissection of the immune microenvironment

**Figure 7:**
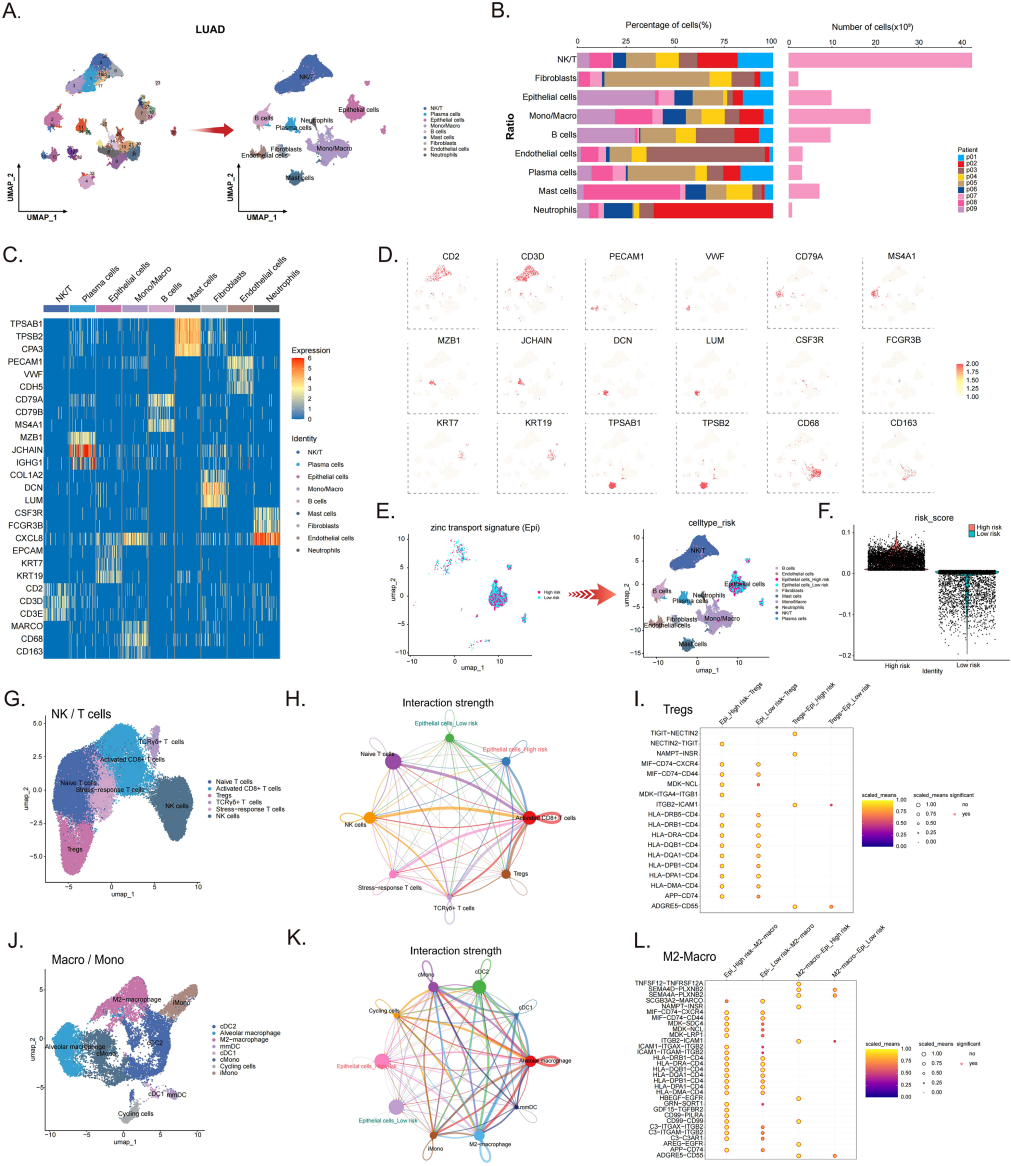
Single-cell dissection of the immune microenvironment. **(A)** UMAP projection showing major cell types in the TME. **(B)** Compositional proportions and abundance of identified cell types. **(C)** Heatmap of cell type-specific marker gene expression patterns. **(D)** UMAP visualization of canonical marker genes validating cell annotations. **(E, F)** Stratification of epithelial cells into HR and LR groups based on risk scores. **(G)** Re-clustering of T/NK cells identifies functional immune subsets. **(H)** Cell-cell communication networks between epithelial subgroups and T/NK cells. **(I)** Key ligand-receptor pairs mediating Treg interactions with high- and low-risk epithelial cells. **(J)** Re-clustering of macrophages identifies functional immune subsets. **(K)** Cell-cell communication networks between epithelial subgroups and macrophages. **(L)** Key ligand-receptor pairs mediating macrophages interactions with high- and low-risk epithelial cells.

Based on the previous immune infiltration analysis suggesting a heightened immunosuppressive status in the HR group, we performed scRNA-seq on 9 LUAD samples to further investigate the underlying mechanisms. UMAP dimensionality reduction identified and annotated the major cell types within the tumor microenvironment (**Figure 7A**), and illustrated their compositional proportions and abundance (**Figure 7B**). Heatmap analysis demonstrated unique marker gene expression profiles for each cell cluster (**Figure 7C**). The spatial expression patterns of canonical markers, including CD3D (T cells), KRT7 (epithelial cells), and CD68 (macrophages), among others, on UMAP plots further validated the reliability of cell annotations (**Figure 7D**). To investigate the applicability of our risk model at the single-cell level, epithelial cells were further stratified into HR and LR groups according to their risk scores (**Figures 7E, F**). Re-clustering of T/NK cells identified functional subsets such as CD4^+^ T cells, Tregs, and NK cells (**Figure 7G**). Cell communication analysis indicated that HR epithelial cells exhibited stronger interaction networks with Tregs compared to their low-risk counterparts (**Figures 7H, I**). Furthermore, macrophage subpopulation analysis revealed close interactions between HR epithelial cells and M2-type macrophages (**Figures 7J-L**). Collectively, these single-cell findings suggest that HR tumor cells may actively recruit and interact with both Tregs and M2 macrophages, thereby contributing to the establishment of an immunosuppressive microenvironment.

### 3.9 Correlation analysis of risk score with immunomodulators and chemokines

**Figure 8:**
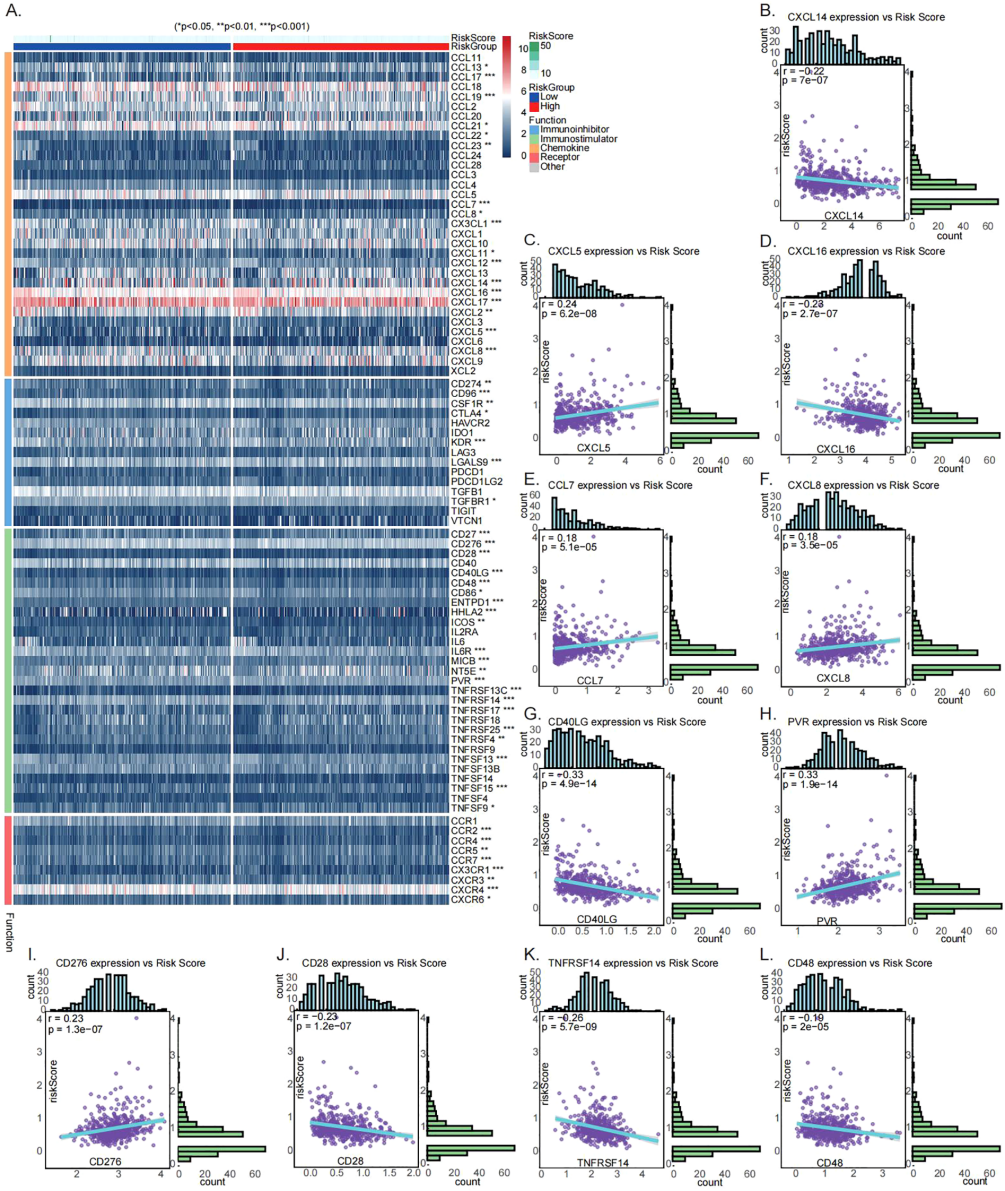
Correlation analysis of risk score with immunomodulators and chemokines. **(A)** Heatmap showing the correlation between risk score and immunomodulators and chemokines. **(B–L)** Scatter plots with regression lines illustrating associations between risk score and the expression levels of *CXCL14*, *CXCL5*, *CXCL16*, *CCL7*, *CXCL8*, *CD40LG*, *PVR*, *CD276*, *CD28*, *TNFRSF14*, and *CD48*.

Immunomodulators and chemokines serve as pivotal drivers of tumorigenesis and cancer progression by mediating immune evasion, remodeling the TME, and promoting metastatic dissemination. As these molecules represent critical therapeutic targets, this study systematically examined the relationship between risk stratification and these immune regulatory factors. Correlation analyses between the risk score and selected immunomodulators and chemokines were visualized via heatmap (**Figure 8A**), revealing significant associations. The risk score correlated with several immune checkpoints (e.g., *CD274*, *CD96*, *CTLA4*, *CSF1R*), immune activators (e.g., *CD27*, *CD28*, *CD276*, *CD40LG*), chemokines (e.g., CCL17, *CCL19*, *CCL7*, *CXCL14*), and chemokine receptors (e.g., *CCR2*, *CCR4*, *CCR7*). Further analysis showed positive correlations between the risk score and *CXCL5*, *CCL7*, *CXCL8*, *PVR*, and *CD276*, while negative correlations were observed with *CXCL14*, *CXCL16*, *CD40LG*, *CD28*, *TNFRSF14*, and *CD48* (**Figures 8B-L**). These associations highlight the immunological distinctions between risk groups and offer a theoretical foundation for developing risk-adapted immunotherapeutic approaches in LUAD.

### 3.10 Chemotherapeutic agent sensitivity profiling

**Figure 9:**
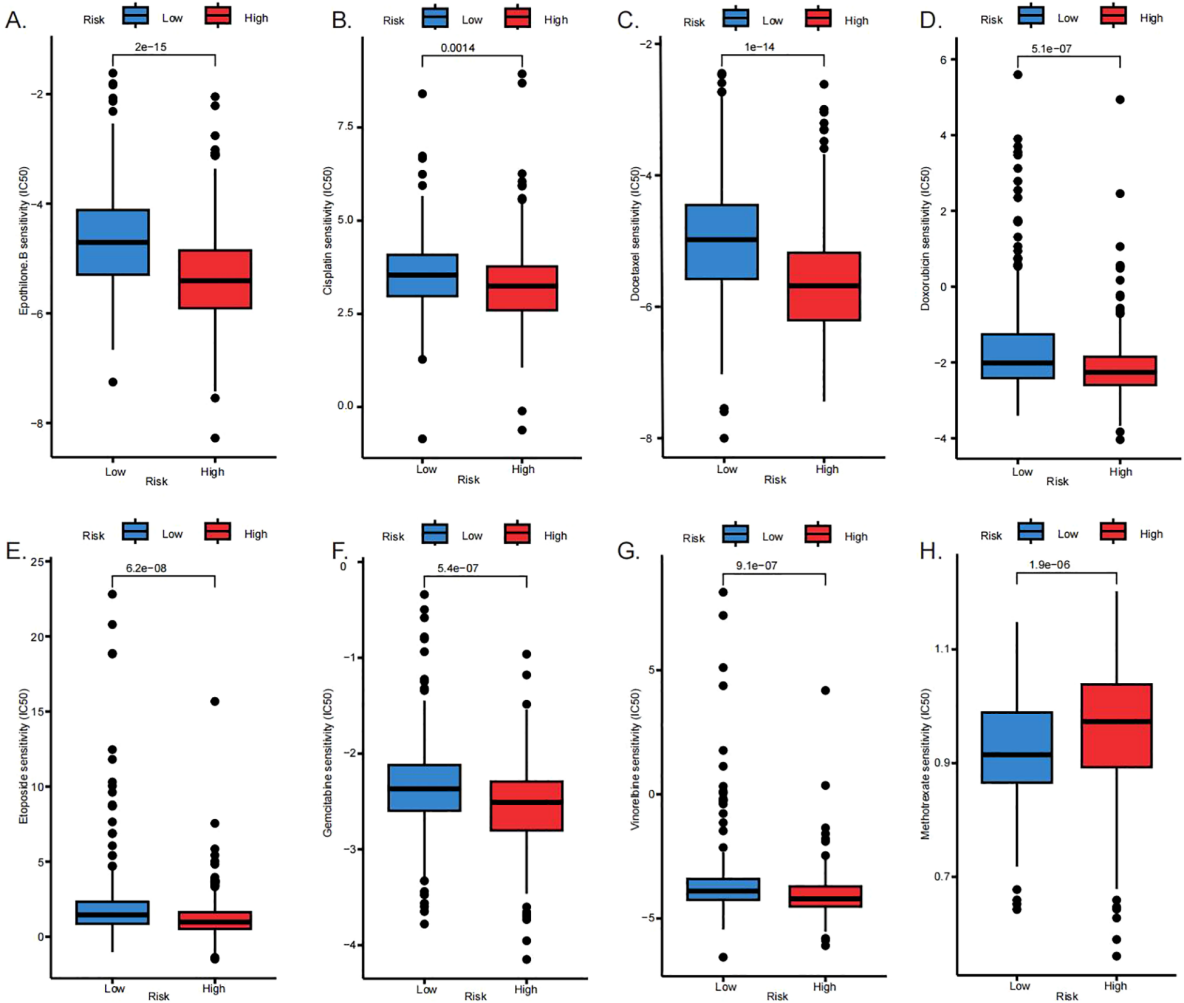
Chemotherapeutic agent sensitivity profiling. Sensitivity responses to individual chemotherapeutic agents are shown: **(A)** Epothilone B, **(B)** Cisplatin, **(C)** Docetaxel, **(D)** Doxorubicin, **(E)** Etoposide, **(F)** Gemcitabine, **(G)** Vinorelbine, and **(H)** Methotrexate.

Given the immune microenvironment characteristics of HR patients, particularly their pronounced resistance to immune checkpoint inhibitors, we therefore conducted drug sensitivity analyses to compare the half-maximal IC50 values of various chemotherapeutic agents across different risk subgroups. The objective was to assess differences in chemoresistance and guide treatment strategies for HR individuals. The results indicated that the HR group displayed significantly greater sensitivity to several agents, including Epothilone B, Cisplatin, Docetaxel, Doxorubicin, Etoposide, Gemcitabine, and Vinorelbine (**Figures 9A-G**). In comparison, Methotrexate was identified as a potentially effective chemotherapeutic option for LR patients (**Figure 9H**).

### 3.11 Risk stratification guides targeted therapy strategies for LUAD

Mutational analysis revealed that HR patients showed elevated tumor mutational burden, with driver mutations primarily enriched in genes involved in the regulation of genomic stability, including *TP53*, *TTN*, *MUC16*, *CSMD3*, and *RYR2* (**Supplementary Figure S4A**). Drug sensitivity profiling showed that LR patients were more responsive to the mTOR inhibitor rapamycin (**Supplementary Figure S4B**) and the EGFR inhibitor erlotinib (**Supplementary Figure S4C**). HR patients exhibited greater sensitivity to a range of agents, including multi-kinase inhibitors (sorafenib, sunitinib), BCR-ABL/PDGFR inhibitors (imatinib), ALK/MET inhibitors (TAE684, CH5424802), and epigenetic regulators (cyclopamine, FTI-277), suggesting that their aggressive disease phenotype may be linked to dysregulated kinase signaling and epigenetic alterations (**Supplementary Figures S4D-J**). Other compounds, such as VX-680, midostaurin, PF-562271, PHA-665752, and MG-132, also showed increased efficacy in this group (**Supplementary Figures S4K-O**).

### 3.12 *In vitro* functional validation of SLC16A3 and EGR2 in lung adenocarcinoma

**Figure 10:**
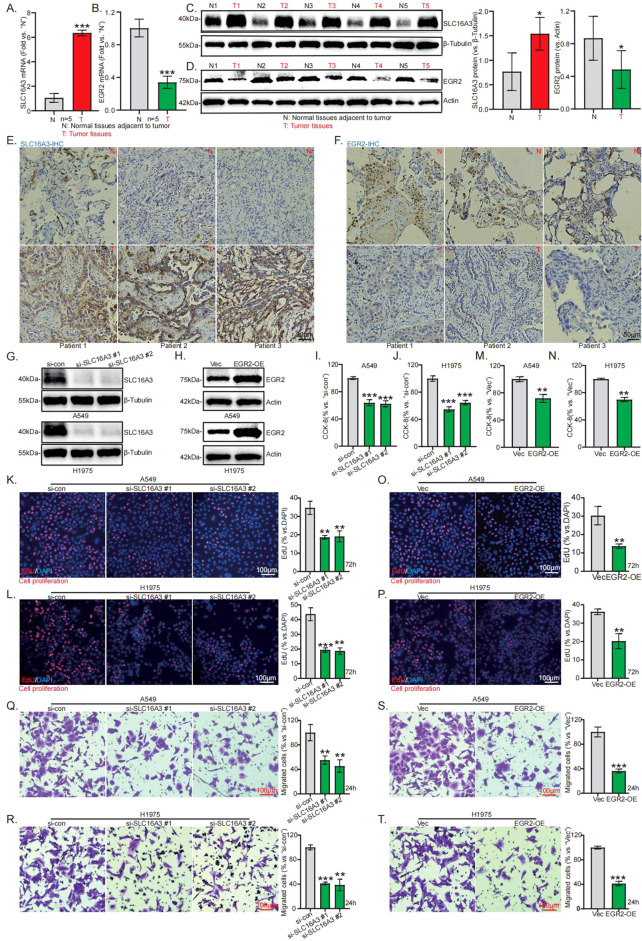
*In Vitro* Functional Validation of SLC16A3 and EGR2 in Lung Adenocarcinoma​. qRT-PCR and Western blotting were applied for testing the expression levels of SLC16A3 **(A, C)** and EGR2 **(B, D)** in lung adenocarcinoma tissues and adjacent normal tissues. Representative immunohistochemical staining images of SLC16A3 **(E)** and EGR2 **(F)** in LUAD tissues and adjacent normal tissues. The intervention efficiency of SLC16A3 knockdown (si-SLC16A3#1, si-SLC16A3#2) and EGR2 overexpression (EGR2-OE) was validated by Western blotting **(G, H)**. CCK-8 assay measured cell proliferation after SLC16A3 depletion **(I, J)** or EGR2 overexpression **(M, N)**. EdU assay evaluated the proliferative capacity of A549 and H1975 cells after SLC16A3 knockdown **(K, L)** or EGR2 overexpression **(O, P)**. Cell migration were measured by “Transwell” assays **(Q-T)**. (*p < 0.05, **p < 0.01, ***p < 0.001).

Given that this model shows significant association with lung adenocarcinoma prognosis, the independent risk genes may critically regulate malignant tumor phenotypes. As the functional roles of the two representative signature genes *SLC16A3* and *EGR2* in lung adenocarcinoma progression remain incompletely elucidated, we selected them for expression and functional validation using a series of *in vitro* assays. Both qRT-PCR (**Figures 10A, B**) and Western blot (**Figures 10C, D**) analyses revealed significant upregulation of SLC16A3 and downregulation of EGR2 in tumor tissues compared to adjacent normal tissues, which was corroborated by immunohistochemistry (**Figures 10E, F**). To investigate their biological functions, we separately performed SLC16A3 knockdown and EGR2 overexpression in lung adenocarcinoma cells, with transfection efficiency confirmed by Western blotting (**Figures 10G, H**). CCK-8 (**Figures 10I, J**) and EdU (**Figures 10K, L**) assays demonstrated that SLC16A3 knockdown significantly inhibited proliferation in A549 and H1975 cells versus controls. EGR2 overexpression similarly suppressed proliferation (**Figures 10M-P**). Furthermore, “Transwell” migration assays showed that SLC16A3 knockdown reduced cellular migration (**Figures 10Q, R**), while EGR2 overexpression likewise inhibited migration (**Figures 10S, T**). These results demonstrate that SLC16A3 acts as an oncogene by promoting proliferation and migration, while EGR2 functions as a tumor suppressor. Both modulating these genes attenuated these malignant behaviors *in vitro*, providing functional validation that aligns with and strengthens the reliability of our prior bioinformatic analyses.

### 3.13 Downregulation of SLC16A3 reduces cisplatin sensitivity of LUAD cells

**Figure 11:**
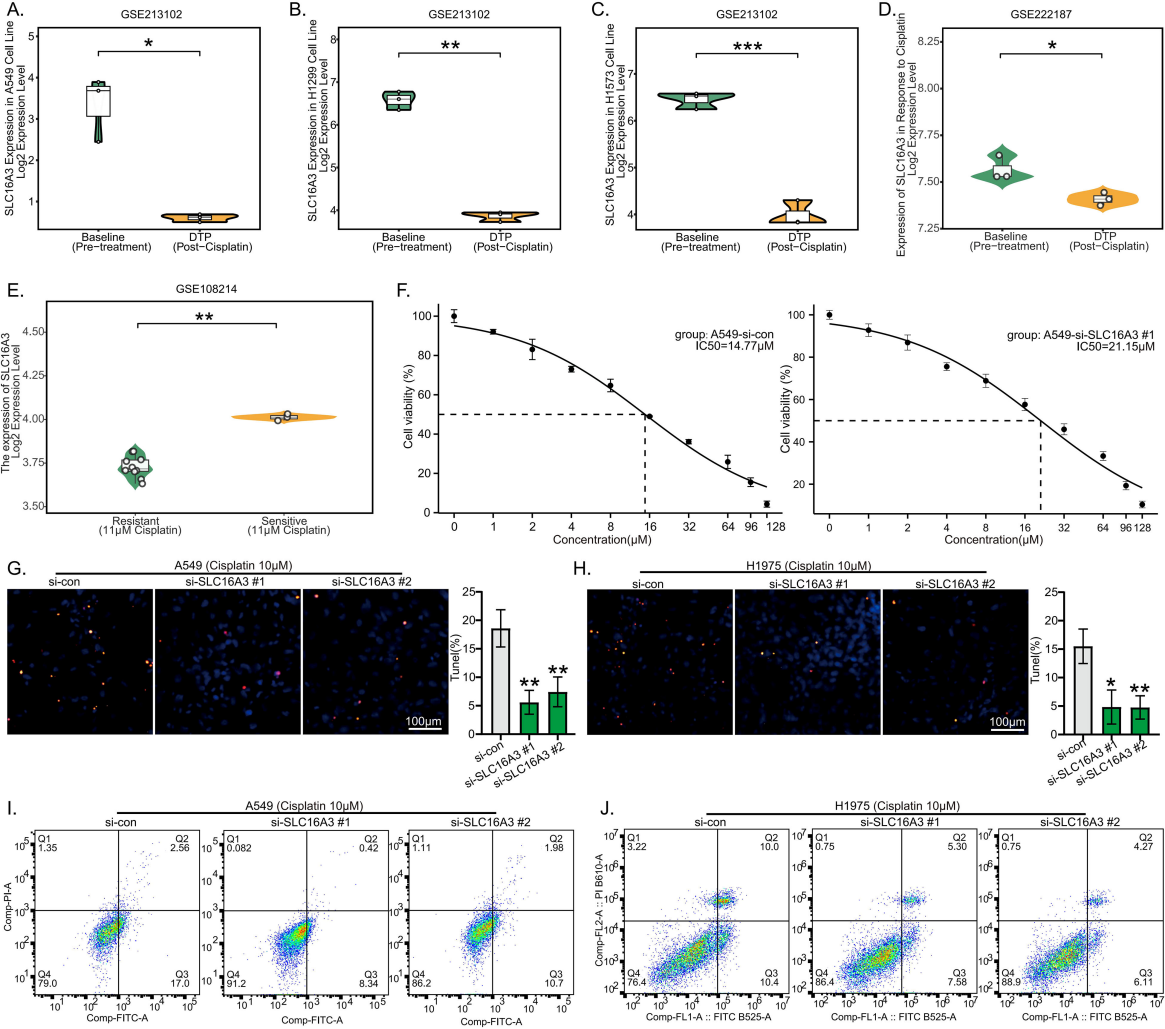
Downregulation of SLC16A3 reduces cisplatin sensitivity of LUAD cells. *SLC16A3* expression in cisplatin-sensitive versus resistant LUAD cell lines was assessed using GSE213102 **(A–C)**, GSE222187 **(D)**, and GSE108214 **(E)** datasets. **(F)** CCK-8 assay for cell viability and IC50 calculation in control and SLC16A3-knockdown A549 cells after 24-hour treatment with a cisplatin concentration gradient. Evaluation of apoptosis by TUNEL staining **(G, H)** and Annexin V/PI flow cytometry **(I, J)**. (*p < 0.05, **p < 0.01, ***p < 0.001).

Prior drug sensitivity analysis indicated that the HR group may be more sensitive to cisplatin (**Figure 9B**). Given that cisplatin is a first-line chemotherapeutic agent for lung adenocarcinoma, we further investigated the relationship between the risk gene *SLC16A3* and cisplatin sensitivity. Analysis of the GSE213102 and GSE222187 datasets revealed that *SLC16A3* expression was significantly lower in multiple cisplatin-resistant lung adenocarcinoma cell lines compared to their corresponding parental cells (**Figures 11A-D**). Similarly, in the GSE108214 dataset, *SLC16A3* expression was also higher in cisplatin-sensitive lung adenocarcinoma cell lines (**Figure 11E**). At the cellular level, knockdown of SLC16A3 in A549 cells resulted in a markedly increased IC50 for cisplatin, suggesting reduced cisplatin sensitivity (**Figure 11F**). Furthermore, in A549 and H1975 cells with SLC16A3 knockdown and treated with 10 μM cisplatin for 24 hours, both TUNEL staining and flow cytometric analysis demonstrated that SLC16A3 downregulation significantly attenuated cisplatin-induced apoptosis compared to the control group (**Figures 11G–J**).

## 4 Discussion

The incidence of LUAD continues to rise, contributing to a growing global patient burden (22). Despite progress in prevention, early detection and treatment, the overall prognosis remains poor (23). Consequently, further exploration of novel and robust prognostic biomarkers is critically needed to improve patients’ overall survival. Zinc homeostasis network-related genes, as key regulators of zinc homeostasis, contribute to intracellular zinc imbalance through aberrant expression and are implicated in the malignant progression of lung cancer (17, 24). While most research has focused on the effects of individual dysregulated genes in tumor progression, more comprehensive models that consider multiple risk genes provide a more robust approach for investigating the collective roles of zinc homeostasis network-related genes in LUAD pathogenesis. Thus, identifying key molecular biomarkers related to zinc homeostasis network and understanding their involvement in disease progression is essential.

In this study, we systematically identified 60 zinc homeostasis network-related DEGs in LUAD, which form significant interaction networks and are predominantly enriched in oncogenic pathways such as PPAR and HIF-1 signaling. This implies that dysregulation of the zinc homeostasis network contributes to LUAD progression through metabolic reprogramming and hypoxia adaptation, consistent with known roles of zinc in energy metabolism and hypoxia (25–28). Through an integrated approach incorporating LASSO regression, univariate Cox analyses, and multivariate Cox analyses, a five-gene prognostic signature was established. Risk stratification demonstrated that HR patients had significantly reduced overall survival and higher mortality rates. The risk score was strongly correlated with advanced T stage, N stage, and pathological stage. The five-gene risk model demonstrated consistent discriminative ability and prognostic patterns across the TCGA-LUAD cohort and multiple external validation cohorts, supporting its stable and reproducible performance. Based on these findings, we propose this model as an auxiliary tool for prognostic evaluation and risk stratification. In clinical practice, quantifying the expression of these five genes in surgical or biopsy specimens generates an individualized risk score that estimates prognostic risk. Moreover, integrating this risk score with clinicopathological features into a nomogram improved the overall discriminative performance and clinical net benefit compared to using clinical variables alone. Our results indicate that the five-gene risk score complements clinical assessment by enabling finer prognostic stratification and providing a valuable reference for personalized treatment decisions, rather than serving as a stand-alone classification tool. GSEA further revealed that HR tumors exhibit features of metabolic reprogramming, including activated glycolysis and HIF-1 signaling, which are established drivers of malignancy and immune evasion in LUAD (29–31).

Significant evidence has shown an intimate relationship between zinc homeostasis network and tumor immunology (32, 33). Using multiple algorithms, our immune profiling revealed significant correlations between risk score and the infiltration of various immune cell populations, including B and T cells, along with macrophages. The LR group exhibited an immunologically active TME, characterized by elevated infiltration of APCs, such as aDCs and iDCs, together with a higher presence of tumor-infiltrating lymphocytes. In contrast, the HR group displayed distinct immunosuppressive traits, including elevated TIDE scores predicting resistance to immune checkpoint inhibitors, and significantly reduced lymphocyte infiltration. Our study further employed single-cell analysis to reveal more active intercellular communication networks between HR tumor cells and immunosuppressive cells, including Tregs and M2-type macrophages. This suggests that HR tumor cells may actively recruit and interact with these immunosuppressive populations. Extensive evidence indicates that the cellular composition and functional state of the TME significantly influence the efficacy of immunotherapy (34, 35). Specifically, Tregs can impair the anti-tumor function of effector T cells through direct contact or the secretion of inhibitory cytokines such as IL-10 and TGF-β (36), while M2-type macrophages promote angiogenesis and suppress T-cell activity by releasing mediators like VEGF and arginase (37). Recent studies have further confirmed that Tregs and M2-type macrophages together constitute key immunosuppressive hubs within the TME, which collectively attenuate the effector functions of infiltrating CD8^+^ T cells and other antitumor immune cells, thereby inhibiting tumor cell killing and limiting the efficacy of immune-based therapies (38–40). Therefore, the synergistic interaction between HR tumor cells and immunosuppressive cells is likely an important mechanism underlying enhanced immune escape and increased disease aggressiveness in HR patients. These insights highlight the importance of targeting immunosuppressive pathways in the development of novel therapeutic strategies for LUAD patients with high-risk profiles. Further analysis of immunoregulatory molecules revealed that the risk score was positively associated with the expression levels of CXCL5, CCL7, CXCL8, PVR, and CD276. Previous reports have shown that these molecules play critical roles in creating an immunosuppressive microenvironment through immune cell recruitment regulation and direct suppression of effector immune cell functions (30, 41–44). In comparison, immunostimulatory molecules such as CXCL14, CXCL16, CD40LG, CD28, and TNFRSF14 displayed negative correlations with risk score. These molecules promote anti-tumor immunity by recruiting effector cells such as NK cells and CD8+ T cells, activating antigen-presenting cells, and directly inhibiting tumor progression (45–50). Together, these findings offer novel insights into the mechanisms through which zinc homeostasis network-related DEGs regulate the immune microenvironment in LUAD.

Based on drug sensitivity profiling, we propose a risk-stratification-guided therapeutic approach. LR patients may benefit from targeted therapies including mTOR inhibitors (e.g., rapamycin) and EGFR inhibitors (e.g., erlotinib), which display greater efficacy in this subgroup. In contrast, HR patients demonstrate increased sensitivity to multi-target kinase inhibitors (e.g., sorafenib, sunitinib) and epigenetic regulators (e.g., cyclopamine). The HR group also shows increased responsiveness to conventional chemotherapeutic agents such as cisplatin and gemcitabine, likely due to compromised DNA repair mechanisms associated with genomic instability (51).

A body of evidence has indicated that the five genes in our model are all involved in the zinc homeostasis network (52–56). In LUAD, they function as a central hub within this network, and their synergistic dysregulation drives disease progression by impacting metabolic reprogramming and immune microenvironment remodeling. Among the five risk genes identified, the functions of LDHA, KLF4, and CAV1 in the onset and progression of LUAD have been relatively well studied (57–59). In contrast, the roles of SLC16A3 and EGR2 in LUAD are relatively limited. Available evidence indicates that SLC16A3, as a member of the monocarboxylate transporter family, promotes immune evasion and metastasis in various tumors by mediating lactate efflux to maintain tumor microenvironment acidification (60, 61). EGR2 is a transcription factor that can restrict tumor growth by regulating tumor suppressor pathways like PTEN (62), but its precise function in LUAD is unknown. To validate their biological functions, we performed systematic *in vitro* assays. We found that SLC16A3 was significantly upregulated and EGR2 was downregulated in LUAD tissues at both mRNA and protein levels. Further functional experiments revealed that knocking down SLC16A3 or overexpressing EGR2 could significantly inhibit the proliferation and migration of LUAD cells. These results confirm that SLC16A3 acts as an oncogene and EGR2 as a tumor suppressor in LUAD, aligning with their identification as key risk genes in our bioinformatic analysis. Moreover, we further discovered that the risk gene *SLC16A3* may be involved in regulating the response of LUAD cells to cisplatin. Analysis of public datasets revealed that *SLC16A3* expression is higher in cisplatin-sensitive lung adenocarcinoma cells. Moreover, knocking down SLC16A3 in lung adenocarcinoma cells attenuated cisplatin-induced apoptosis. These findings align with the trend observed in our drug sensitivity analysis, where the high-risk group appeared to derive greater benefit from cisplatin treatment, suggesting the potential utility of this five-gene risk model in guiding adjuvant treatment strategy selection.

This study has several strengths. We conducted a comprehensive investigation of zinc homeostasis-associated genes in LUAD and developed a five-gene risk model. The model’s risk score was associated with aggressive clinicopathological features and exhibited consistent prognostic performance across multiple cohorts. Furthermore, because the model includes only five genes, it can be easily combined with clinical characteristics in a nomogram to improve overall predictive performance and clinical net benefit. The signature genes are implicated in oncogenic pathways and immune regulation, suggesting their potential utility in predicting immunotherapy response and guiding risk-based precision therapy. Finally, experimental validation of SLC16A3 and EGR2 underscores the biological relevance of our model. Nevertheless, certain limitations should be acknowledged. The overall predictive accuracy of the model, as indicated by the AUC and C-index, was moderate. This may stem from the inherent biological and clinical heterogeneity of LUAD, the retrospective nature of the public cohorts, and potential sample selection biases. Thus, validation in larger, prospective, multicenter cohorts is still required. Furthermore, functional validation was performed exclusively *in vitro* and focused on two key genes, while *CAV1*, *KLF4*, and *LDHA* were not functionally examined in this study. Elucidating the functions of these genes is a priority for future research to fully uncover the model’s link to tumor progression. Additionally, while our analyses suggest that the signature influences the tumor immune microenvironment via regulatory T cells and M2 macrophages, the precise molecular mechanisms require further investigation.

## 5 Conclusion

A prognostic risk model for LUAD was established based on zinc homeostasis network-related genes, demonstrating significant correlations with clinical stage and with patient survival. HR patients show poorer prognoses and features of an immunosuppressive microenvironment, whereas LR patients display a “hot tumor” phenotype associated with greater immunotherapy responsiveness. Furthermore, risk stratification guides potential therapeutic strategies, revealing differential sensitivities to chemotherapeutic and targeted agents. This model bridges the zinc homeostasis network with actionable precision therapy and may have important implications for personalized treatment and risk assessment in patients, with potential for clinical translation.

## Data Availability

The original contributions presented in the study are included in the article/**Supplementary Material**. Further inquiries can be directed to the corresponding author.

## Supplementary material

Supplementary Figure 1 GO **(A, B)** and KEGG pathway **(C)** enrichment analysis. **(D)** LogFC-weighted enrichment validation.

Supplementary Figure 2 Time-dependent ROC curves of **(A)** CAV1, **(B)** EGR2, **(C)** KLF4, **(D)** LDHA, and **(E)** SLC16A3 for predicting 1-, 3-, and 5-year overall survival.

Supplementary Figure 3 **(A)** GO analysis. **(B)** GSVA and GSEA **(C, D)** between HR and LR groups.

Supplementary Figure 4 **(A)** Mutation site analysis in high- and LR patient groups. Comparative assessment of therapeutic response to targeted agents between the two patient cohorts: **(B)** Rapamycin, **(C)** Erlotinib, **(D)** Sorafenib, **(E)** Sunitinib, **(F)** Imatinib, **(G)** TAE684, **(H)** CH5424802, **(I)** Cyclopamine, **(J)** FTI-277, **(K)** VX-680, **(L)** Midostaurin, **(M)** PF-562271, **(N)** PHA-665752, **(O)** MG-132.
