# Supplementary material for: Integrating zinc homeostasis network and immune landscape: a five-gene prognostic framework for precision oncology in lung adenocarcinoma
Source: Front Immunol. 2026 Jan 8;16:1691179. doi: 10.3389/fimmu.2025.1691179 (PMC12823828; doi:10.3389/fimmu.2025.1691179)

A.

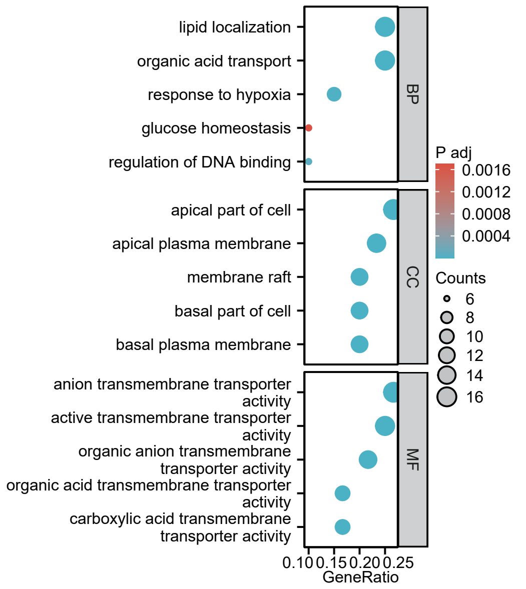

B.

ID	Description
GO:0015849	organic acid transport
GO:0010876	lipid localization
GO:0001666	response to hypoxia
GO:0051101	regulation of DNA binding
GO:0042593	glucose homeostasis
GO:0045177	apical part of cell
GO:0016324	apical plasma membrane
GO:0009925	basal plasma membrane
GO:0045178	basal part of cell
GO:0045121	membrane raft
GO:0008509	anion transmembrane transporter activity
GO:0008514	organic anion transmembrane transporter activity
GO:0022804	active transmembrane transporter activity
GO:0046943	carboxylic acid transmembrane transporter activity
GO:0005342	organic acid transmembrane transporter activity

C.

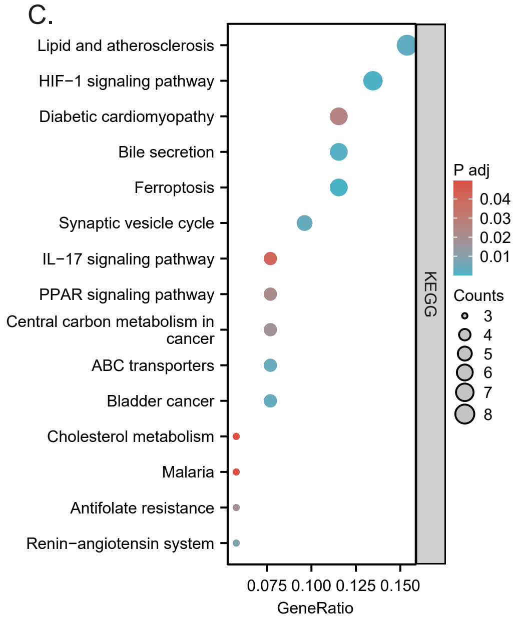

D.

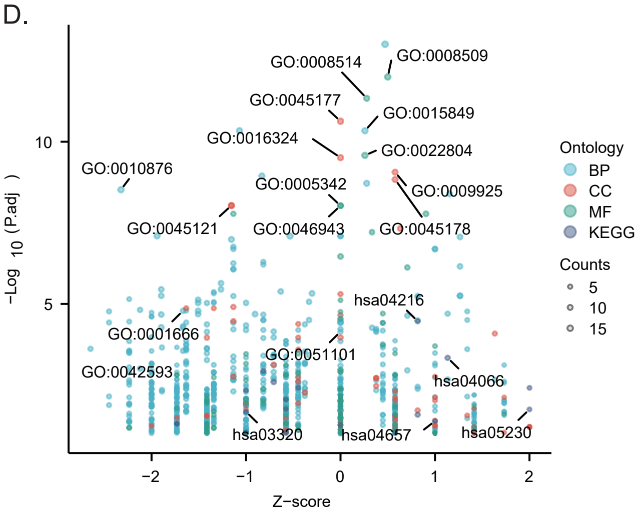

Supplement: Supplementary Figure 1 — GO (A, B) and KEGG pathway (C) enrichment analysis. (D) LogFC-weighted enrichment validation. [file DataSheet1.pdf]
