# Supplementary figures and images for: Integrating zinc homeostasis network and immune landscape: a five-gene prognostic framework for precision oncology in lung adenocarcinoma
Source: Front Immunol. 2026 Jan 8;16:1691179. doi: 10.3389/fimmu.2025.1691179 (PMC12823828; doi:10.3389/fimmu.2025.1691179)

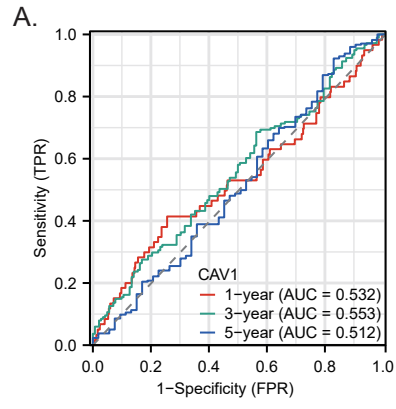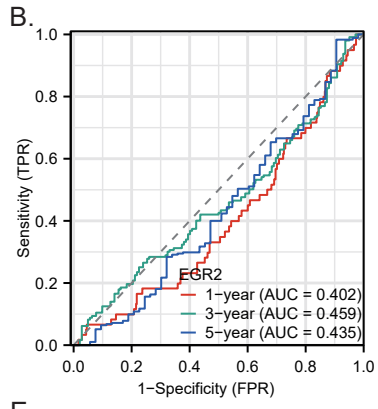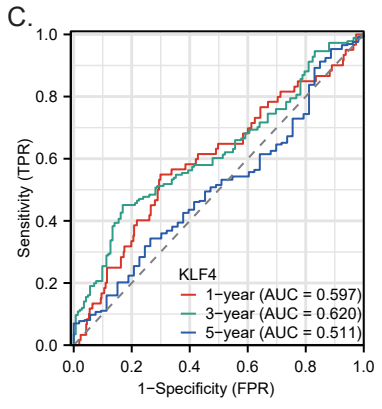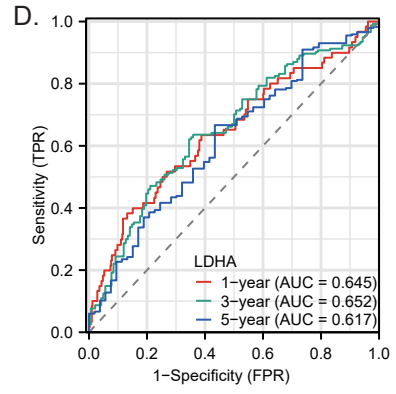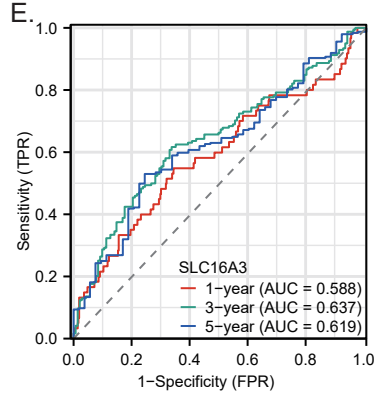

Supplement: Supplementary Figure 2 — Time-dependent ROC curves of (A) CAV1, (B) EGR2, (C) KLF4, (D) LDHA, and (E) SLC16A3 for predicting 1-, 3-, and 5-year overall survival. [file DataSheet2.pdf]

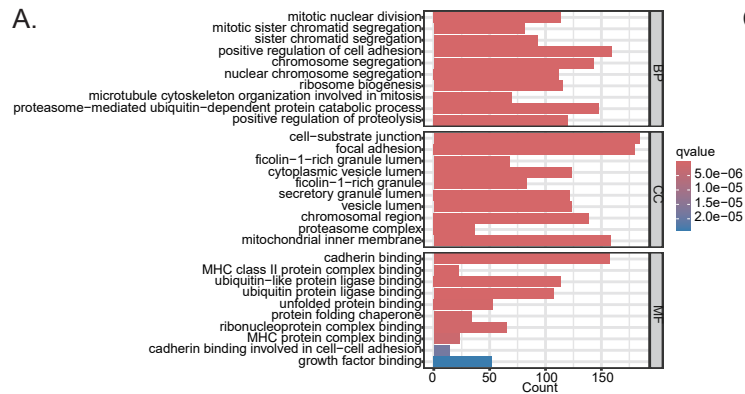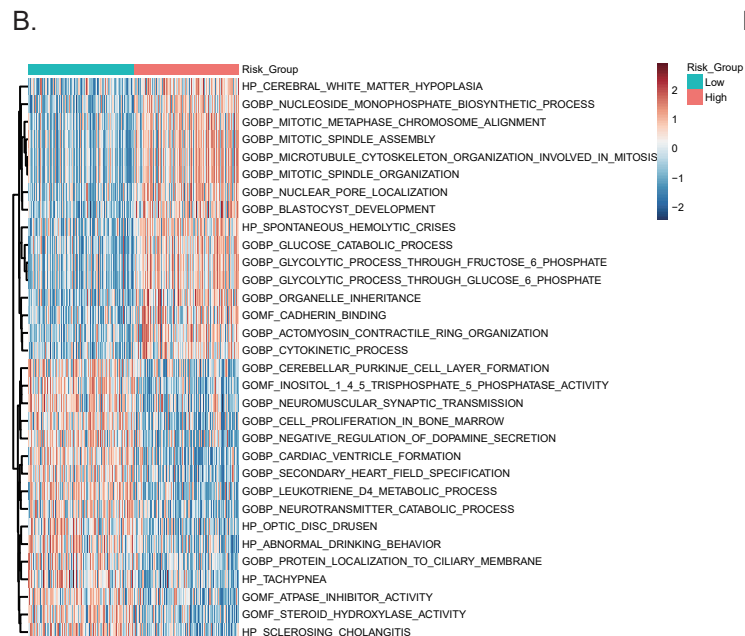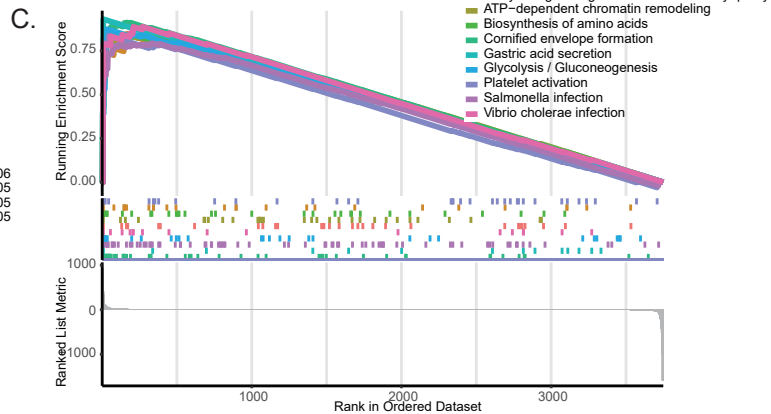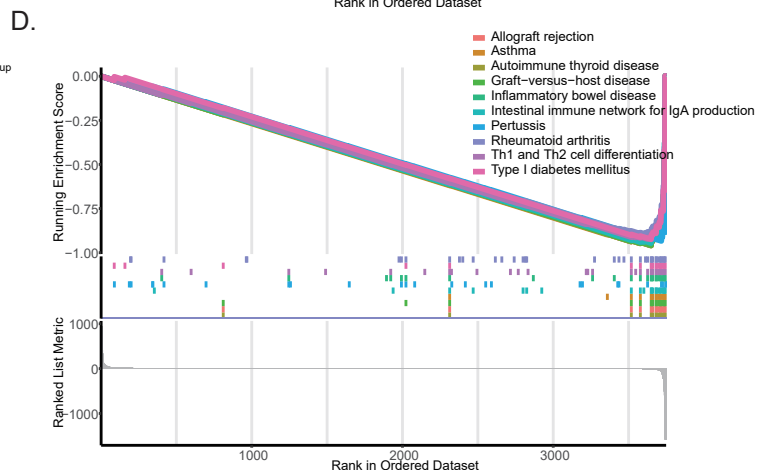

Supplement: Supplementary Figure 3 — (A) GO analysis. (B) GSVA and GSEA (C, D) between HR and LR groups. [file DataSheet3.pdf]

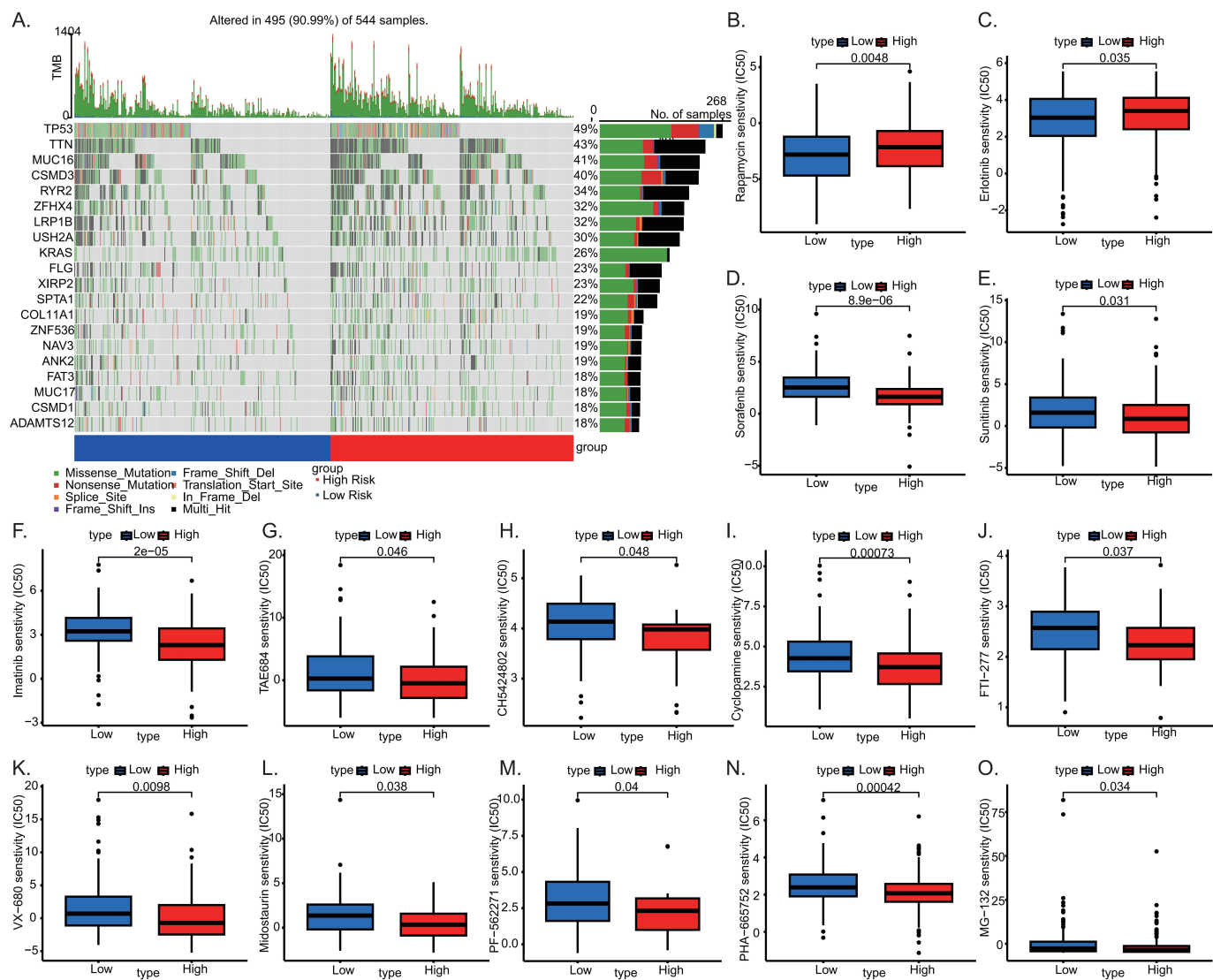

Supplement: Supplementary Figure 4 — (A) Mutation site analysis in high- and LR patient groups. Comparative assessment of therapeutic response to targeted agents between the two patient cohorts: (B) Rapamycin, (C) Erlotinib, (D) Sorafenib, (E) Sunitinib, (F) Imatinib, (G) TAE684, (H) CH5424802, (I) Cyclopamine, (J) FTI-277, (K) VX-680, (L) Midostaurin, (M) PF-562271, (N) PHA-665752, (O) MG-132. [file DataSheet4.pdf]

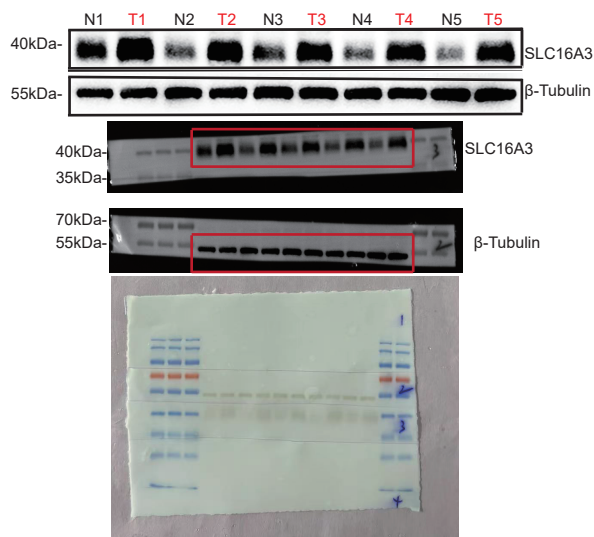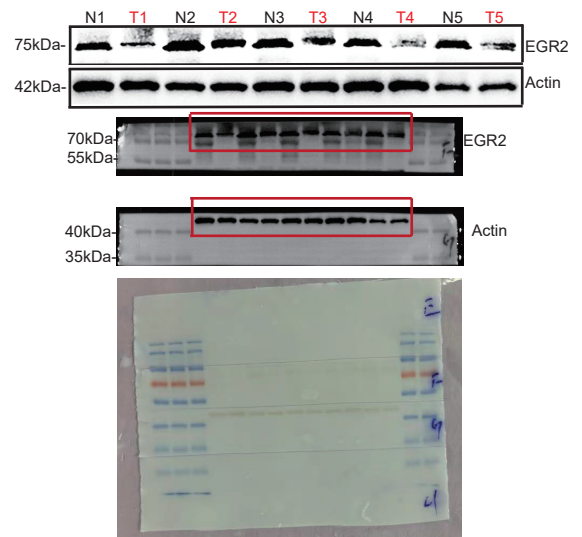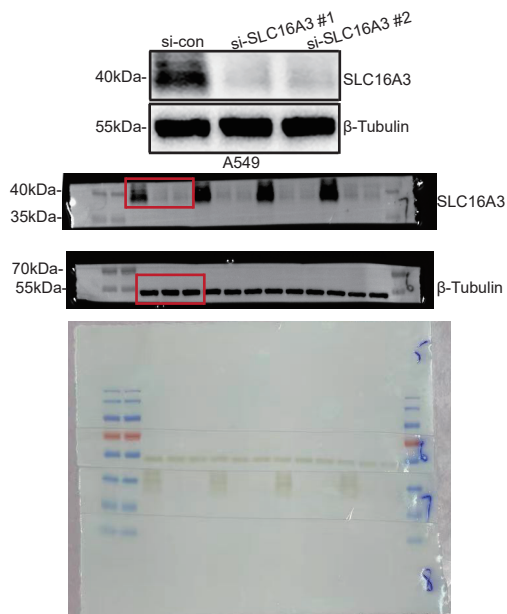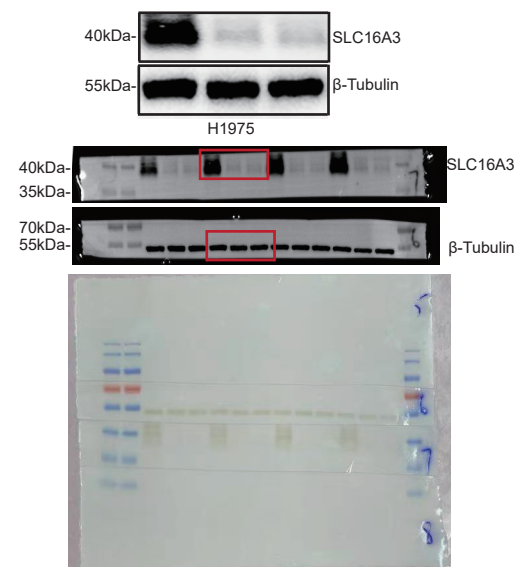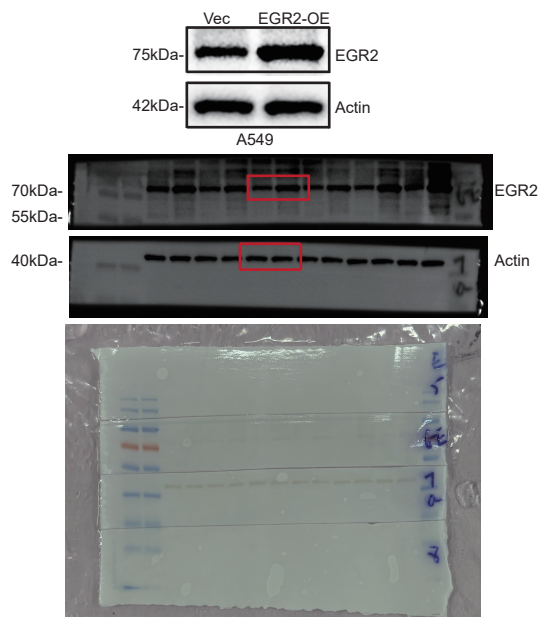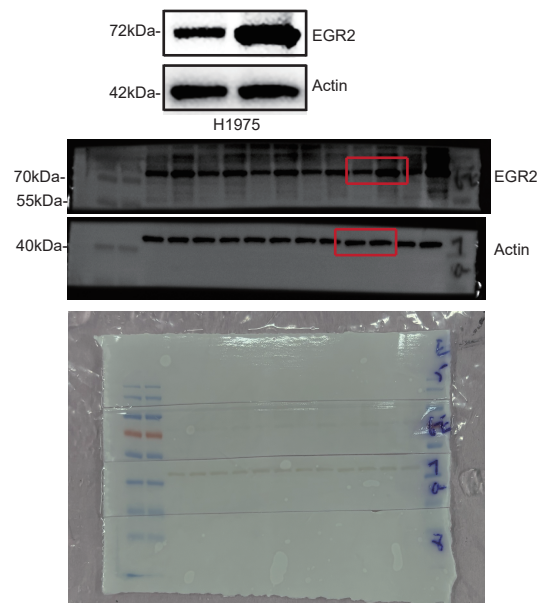

Supplement: Supplementary file 5 [file Image1.pdf]
