# Supplementary material for: Integrating zinc homeostasis network and immune landscape: a five-gene prognostic framework for precision oncology in lung adenocarcinoma
Source: Front Immunol. 2026 Jan 8;16:1691179. doi: 10.3389/fimmu.2025.1691179 (PMC12823828; doi:10.3389/fimmu.2025.1691179)
Supplement: Supplementary file 6 [file Table1.docx]

| Premier | Sequences (5′–3′) |
| --- | --- |
| SLC16A3-F | GCCCTACTCCGTCTACCTCT |
| SLC16A3-R | TGCCGTAGGAGATGCCAAAG |
| EGR2-F | AGTTTGCCCGGAGTGATGAG |
| EGR2-R | CTGTTACTGCTGCACAGGGT |
| GAPDH-F | GGTGTGAACCATGAGAAGTATGA |
| GAPDH-R | GAGTCCTTCCACGATACCAAAG |

Supplementary Table 1. Primer sequences for qRT-PCR analysis
