# Supplementary material for: Integrating zinc homeostasis network and immune landscape: a five-gene prognostic framework for precision oncology in lung adenocarcinoma
Source: Front Immunol. 2026 Jan 8;16:1691179. doi: 10.3389/fimmu.2025.1691179 (PMC12823828; doi:10.3389/fimmu.2025.1691179)
Supplement: Supplementary file 7 [file Table2.docx]

**Supplementary Materials and Methods**

**Functional enrichment analysis:** Enrichment analyses were performed using the “clusterProfiler” and “org.hs.eg.db” R packages to identify key functions via Gene Ontology (GO) and Kyoto Encyclopedia of Genes and Genomes (KEGG) analyses, with statistical significance determined by Benjamini-Hochberg (BH) multiple testing correction (significance thresholds: adjusted p-value < 0.05). GO terms were classified into Biological Process (BP), Molecular Function (MF), and Cellular Component (CC). Gene Set Enrichment Analysis (GSEA) and Gene Set Variation Analysis (GSVA) were utilized for comparison of the potential characteristic signaling pathways changes between high- and low- risk groups.

**Correlation analysis of risk score with immunomodulators and chemokines:** Immunomodulatory and chemokine gene sets were obtained from the TISIDB platform (http://cis.hku.hk/TISIDB/). Associations between the levels of selected immune-related gene expression and the risk score were examined using the “limma” packages in R, and the findings were presented as heatmaps. Scatter plots with regression lines were generated employing “ggplot2,” “dplyr,” and “patchwork” R packages.

**Processing and Cell Communication Analysis of scRNA-seq Data:** Single-cell RNA sequencing (scRNA-seq) data were obtained from the publicly available GEO dataset GSE189357, which includes nine lung adenocarcinoma samples. Raw scRNA-seq data were processed to generate quality-controlled gene expression matrices with the CreateSeuratObject function, retaining genes expressed in at least three cells. Cells with high mitochondrial or erythrocyte gene content (>20%) or abnormal total gene counts (<1000) were excluded to ensure data integrity. Potential doublets were identified and removed using the DoubletFinder package prior to downstream analyses. After rigorous quality control, 97206 cells were retained for further analysis. Data normalization (NormalizeData) and feature scaling (ScaleData) were performed, and highly variable genes were identified using FindVariableFeatures. PCA was conducted, with the optimal number of components determined by JackStraw statistical validation and ElbowPlot variance analysis. Nonlinear dimensionality reduction was achieved using uniform manifold approximation and projection (UMAP). Cell clusters were identified through graph-based clustering, and DEGs between tumor and normal clusters were detected using the FindMarkers function (adjusted p-value < 0.05, |log₂ fold change| > 1.0). To further classify epithelial cells, a risk score was calculated for each cell based on the above formula derived from bulk RNA-seq analysis. Epithelial cells were then divided into HR and LR groups according to their individual risk scores. The CellChat package (v1.6.1) was employed to investigate intercellular communication networks. CellChat analysis was performed based on normalized gene expression data and cluster annotations to infer and compare signaling pathway activities between tumor epithelial and immune cell populations.

**Drug sensitivity analysis:** Differences in drug sensitivity between the high-risk and low-risk groups were assessed by estimating the half-maximal inhibitory concentration (IC50) values utilizing “oncoPredict” and “pRRophetic” packages in R. Intergroup comparisons were conducted using Wilcoxon rank-sum tests with significance set at p< 0.05.

**qRT-PCR assay:**Total RNA was extracted from tissues using an RNA rapid extraction kit, reverse-transcribed into cDNA, and amplified by qRT-PCR in 20 μL reaction volumes prepared with SYBR Green PCR Master Mix (Ribo-Bio, Guangzhou). GAPDH mRNA was used as an internal reference gene for normalization. Relative mRNA expression levels of target genes were calculated using the 2−ΔΔCt method. The gene primer sequences are listed in Supplementary Table 1.

**Western Blotting:**Protein samples (20 μg per lane) were separated on 10% SDS-PAGE gels, electrophoresed at 80 V until samples entered the separating gel, then at 120 V until complete resolution. Proteins were transferred onto PVDF membranes (Millipore, Bedford, MA) at 300 mA for 95 minutes. After blocking with 5% skim milk for 2 hours at room temperature, membranes were sequentially incubated with primary antibodies overnight at 4°C and corresponding secondary antibodies for 2 hours at room temperature. Membranes were washed three times (10 min each) with TBST after each antibody incubation. Protein bands were visualized using ECL chemiluminescent reagent, and quantified with ImageJ. Primary antibodies included: anti-SLC16A3 (1:2000, Proteintech), anti-EGR2 (1:2000, Proteintech), anti-β-tubulin (1:4000, Proteintech), and anti-actin (1:4000, Proteintech).

**Immunohistochemistry (IHC):** Briefly, immunohistochemical staining was conducted on paraffin-embedded tissue sections. Following dewaxing, 3-μm-thick sections were incubated overnight with diluted primary antibodies. After washing with PBS, sections were incubated with secondary antibody for 30 minutes, and images were acquired using an Olympus FSX100 microscope.
